# Two novel cases with PIGQ-CDG: expansion of the genotype–phenotype spectrum and evaluation of GestaltMatcher as a diagnostic tool

**DOI:** 10.3389/fgene.2025.1598602

**Published:** 2025-07-11

**Authors:** Katarína Kušíková, Tzung-Chien Hsieh, Mateja Pfeifer, Christine Fauth, Yoshiko Murakami, Franco Laccone, Daniela Karall, Walter Bonfig, Helen Stewart, Denisa Weis

**Affiliations:** ^1^ Department of Pediatric Neurology, Medical School, Comenius University and National Institute of Children’s Diseases, Bratislava, Slovakia; ^2^ Institute for Genomic Statistics and Bioinformatics, University Hospital Bonn, Rheinische Friedrich-Wilhelms-University Bonn, Bonn, Germany; ^3^ Institute of Medical Genetics, Medical University of Vienna, Vienna, Austria; ^4^ Institute of Human Genetics, Medical University of Innsbruck, Innsbruck, Austria; ^5^ Laboratory of Immunoglycobiology, Research Institute for microbial diseases, Osaka University, Osaka, Japan; ^6^ Department of Pediatrics I, Inherited Metabolic Disorders, Medical University of Innsbruck, Innsbruck, Austria; ^7^ Department of Pediatrics, Klinikum Wels-Grieskirchen, Wels, Austria; ^8^ Oxford Centre for Genomic Medicine, Oxford University Hospitals NHS Foundation Trust, Oxford, United Kingdom; ^9^ Department of Medical Genetics, Kepler University Hospital, School of Medicine, Johannes Kepler University, Linz, Austria

**Keywords:** PIGQ-CDG, *PIGQ* gene, GPI-anchor, GPI-anchor deficiency, MCAHS4, rhabdomyolysis, hyperCKemia, GestaltMatcher

## Abstract

**Introduction:**

The glycosylphosphatidylinositol (GPI) anchor is a glycolipid that anchors proteins to the eukaryotic cell surface. An anchoring process is a posttranslational modification of at least 150 molecules with various functions. Biallelic causal variants in the *PIGQ* gene (OMIM: * 605754) are associated with a type of disorder of glycosylphosphatidylinositol biosynthesis (PIGQ-congenital disorders of glycosylation (CDGs), also called multiple congenital anomalies-hypotonia-seizures syndrome 4 (MCAHS4, OMIM: # 618548). Only 11 patients with this condition have been reported to date.

**Methods:**

We present two novel cases of MCAHS4 with one novel and one already known variant in the *PIGQ* gene, detailed phenotyping, and a review of all published cases so far. We used GestaltMatcher for deep gestalt analysis and investigated its potential use in diagnosing MCAHS4 patients.

**Results:**

In the *PIGQ* gene, we found one novel frameshift variant c.1092dupC, p.(Phe365LeufsTer78) and one missense c.1370T>G, p.(Leu457Arg) already listed in the ClinVar database as a variant of uncertain significance (VUS), whose pathogenicity we proved by a functional study on Chinese hamster ovarian cells. After reviewing all 13 already diagnosed MCAHS4 patients, we found that attacks of rhabdomyolysis induced by a febrile infection were documented only in our patient. Facial dysmorphism (coarse features, anteverted nares, and open mouth) seen in all analyzed MCAHS4 patients seems to be specific. Moreover, GestaltMatcher proved that MCAHS4 patients shared a similar facial phenotype.

**Discussion:**

The present work expands the genotype spectrum by describing a novel causal *PIGQ* variant and validating the pathogenicity of an already-known VUS variant. Because of their life-threatening complications, attacks of rhabdomyolysis should be considered in MCAHS4 patients. GestaltMatcher can be an effective tool in the diagnostic setting of MCAHS4.

## 1 Introduction

The glycosylphosphatidylinositol (GPI)-anchor is a glycolipid that anchors proteins to the eukaryotic cell surface. An anchoring process is a posttranslational modification of at least 150 proteins, including neural and complement regulatory proteins, cell surface antigens, adhesion molecules, and many other molecules with various functions (Kinoshita, 2014). These proteins are active during embryogenesis, immunological response, cell signaling, and neurogenesis (Kinoshita, 2020). Today, more than 31 genes are involved in the GPI-anchor biosynthesis pathway (Synthesis Group and Transamidase+Remodeling Group), of which 25 are responsible for specific human diseases (Carmody et al., 2020; Ng et al., 2024). Disorders of glycosylphosphatidylinositol biosynthesis represent a subgroup of a large group known as congenital disorders of glycosylation (CDGs) (Carmody et al., 2020; Ng et al., 2024). Similar to CDGs, GPI-anchor deficiencies are associated with a broad phenotypic spectrum. However, developmental delay, epilepsy, muscular hypotonia, facial dysmorphism, organ malformations, and skeletal abnormalities are mainly present (Knaus et al., 2019; Carmody et al., 2020).

The *PIGQ* gene (OMIM: * 605754, locus 16p13.3), consisting of 11 exons, encodes the eponymous PhosphatidylInositol Glycan class Q protein (PIGQ protein). This 760-amino-acid polypeptide is a member of the GPI-anchor Synthesis Group genes and is involved in the first step of GPI-anchor biosynthesis (Carmody et al., 2020; Wu et al., 2020). Concretely, the *PIGQ* gene encodes an N-acetylglucosaminyl transferase component. As a part of a molecular complex, its function is to catalyze the transfer of N-acetylglucosamine (GlcNAc) from UDP-GlcNAc to phosphatidylinositol (PI) (Watanabe et al., 1998; Tiede et al., 2001).

According to the OMIM database (Hamosh et al., 2005), biallelic causal variants in the *PIGQ* gene are associated with multiple congenital anomalies-hypotonia-seizures syndrome 4 (MCAHS4, OMIM: # 618548), inherited in an autosomal recessive manner (Tiede et al., 2001). According to Ng et al., 2024, these variants are nosologically named PIGQ-CDG. MCAHS4 typically presents with severe developmental delay, craniofacial dysmorphism, early-onset therapy-resistant epileptic encephalopathy, hypotonia, and premature death (Hamosh et al., 2005). To date, 11 cases of MCAHS4 patients have been reported (Martin et al., 2014; Alazami et al., 2015; Starr et al., 2019; Johnstone et al., 2020; Zanni et al., 2022), of which only two were published with known genotypes; their phenotypic data have remained limited (Martin et al., 2014; Alazami et al., 2015).

We present the case of two new MCAHS4 patients whose genotype and phenotype were compared with already published cases. The pathogenicity validation of the missense variant L457R was performed by a functional study on Chinese hamster ovary cells (CHO). For the first time, we broadened the PIGQ-CDG phenotype spectrum by attacks of rhabdomyolysis, that is, muscle breakdown with the release of intracellular contents into the blood, triggered by an infection in one of the presented cases where hyperCKemia is detected. Finally, we analyzed facial features in MCAHS4 patients. Facial gestalt seems unique for these patients and suitable for deep gestalt analysis. Therefore, we evaluated its potential diagnostic benefit by GestaltMatcher (Hsieh et al., 2022).

## 2 Materials and methods

### 2.1 Patients involved in the study

Our study involved two novel cases and 11 already reported MCAHS4 patients (Martin et al., 2014; Alazami et al., 2015; Starr et al., 2019; Johnstone et al., 2020; Zanni et al., 2022). The first novel patient (P1) was from the Department of Pediatrics Klinikum Wels-Grieskirchen in Austria, and the second (P2) was from the Department of Pediatrics I, Inherited Metabolic Disorders, Medical University of Innsbruck in Austria. After obtaining written informed consent from the patient’s parents, blood samples, laboratory and clinical data collection, and photodocumentaries were obtained. Both patients underwent a detailed clinical examination. The studies involving human participants were reviewed and approved by the local Ethics Committee (available in Supplementary Information). Information about already published patients was gained retrospectively. The obtained information about the genotype and phenotype of the involved subjects was analyzed by descriptive statistics.

### 2.2 Genetic analysis

Genetic analysis of P1 and P2 was performed on DNA isolated from the leukocytes of peripheral blood samples using the standardized routine in-house protocol. Both patients underwent karyotyping and array CGH analysis, followed by whole-exome sequencing (WES). The human reference genome GRCh37/hg19 was used for all exome alignments. Detailed WES protocols can be found in Supplementary Materials. A uniform transcript NM_004204.3 was used to report variants detected in the *PIGQ* gene, and variants were classified according to ACMG recommendations (Richards et al., 2015).

### 2.3 Functional study for the missense variant L457R in the *PIGQ* gene in P2

A deleterious effect of the VUS missense variant c.1370T>G, p.(Leu457Arg) in the *PIGQ* gene (found in P2) already listed in the ClinVar database was investigated on Chinese hamster ovarian (CHO) cells, inspired by a published protocol (Martin et al., 2014), and P2 fibroblasts. *PIGQ*-deficient CHO cells (CHO10.2.1) were transiently transfected with wild-type or Y400del mutant or L457R mutant *PIGQ* cDNA with FLAG-tag at the N-terminus, driven by the strong SRα promoter (pME F-hPIGQ), a weak thymidine kinase promoter (pTK), or a minimum TATA box promoter (pTA). Two days later, cells were stained with anti-CD59 (5H8), -uPAR (VIM5, Biolegend, San Diego, CA), and DAF (IA10) and analyzed by flow cytometry. The patient’s fibroblasts were immortalized by transduction of telomerase reverse transcriptase (Tert) expressing a retrovirus vector and were stained with antibodies for the GPI-APs, CD59, DAF, uPAR, FLAER (CEDARLANE, Canada), CD90 (5E10 Biolegend), and CD109 (W7C5, Biolegend). To confirm that the reduction was caused by PIGQ deficiency, we transduced with retrovirus empty vector or wild-type *PIGQ* cDNA expressing vector and stained for flow cytometry (Supplementary Figure S1; Fibroblasts (TERT)). Lysates were applied to SDS-PAGE, and Western blotting was performed using an anti-FLAG antibody (M2, Sigma, St. Louis, MO) to detect PIGQ expression and an anti-GAPDH antibody (6C5, Life Technologies, CA) to detect endogenous GAPDH as a loading control (Supplementary Figure S2; Fibroblasts). We transiently transfected the wild-type or mutant pME FLAG PIGQ in HEK 293 cells. Two days later, lysates were applied to SDS-PAGE, and Western blotting was performed using an anti-FLAG antibody (M2, Sigma, St. Louis, MO) to detect PIGQ expression and anti-GAPDH (6C5, Life Technologies, CA) to detect endogenous GAPDH as a loading control. Transfection efficiency was monitored by Luciferase activities using a Luciferase assay kit (Promega, Madison, WI) (Supplementary Figure S3; Western blot).

### 2.4 Computational facial analysis by GestaltMatcher

Computational facial analysis by GestaltMatcher (Hsieh et al., 2022; Hustinx et al., 2023; Lesmann et al., 2024) was used for craniofacial features evaluation. We encoded each image into twelve 512-dimensional vectors using model ensemble and test-time augmentation. These vectors, known as facial embeddings, represent key facial features in a high-dimensional numerical space where similar faces are located closer together. To quantify similarity between individuals, we computed the cosine distance between embeddings: a smaller cosine distance indicates greater facial similarity, suggesting proximity within the phenotype space. For each image pair, we averaged the cosine distances across the 12 embeddings to obtain a robust measure of phenotypic proximity. We initiated a cohort-level analysis to verify the similarity among individuals with the *PIGQ* gene. This was followed by an individual-level analysis, which allowed us to delve deeper into these similarities and draw more nuanced conclusions.

## 3 Results

### 3.1 Genotype in MCAHS4 patients

**FIGURE 1:**
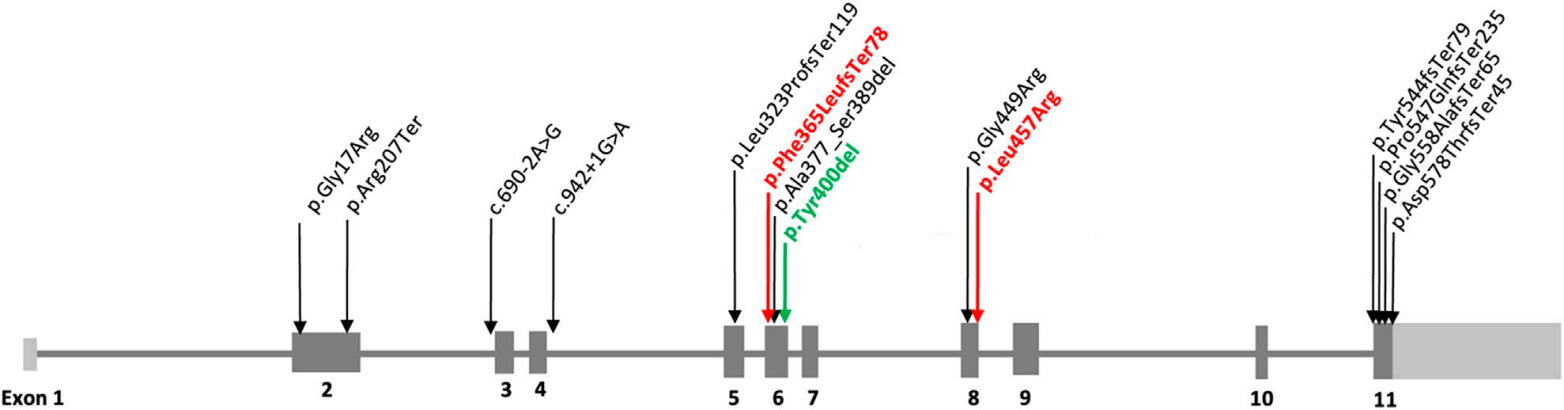
Variants identified in the *PIGQ* gene (NM_004204.3). The schematic figure of the *PIGQ* gene summarizes all published variants, including the new one c.1092dupC, p.(Phe365LeufsTer78). The variants that we describe in our work are highlighted in bold red, and the known recurrent variant is highlighted in bold green.

Genotype information was available in all 13 MCAHS4 patients (n = 13) from 12 unrelated families and different ancestries. Subjects P5 (St3a) and P6 (St3b) are siblings. We have unified the previously published variants according to the NM_004204.3 transcript and re-evaluated their pathogenicity according to the ACMG classification (Richards et al., 2015) (Table 1). Variants are distributed through the *PIGQ* gene, mainly in exons 2, 5, 6, 8, and 11, of which exon 6 is mostly affected (Figure 1). The frequent variant is in-frame pathogenic (PP5+PM4+PM2) deletion c.1199_1201del, p.(Tyr400del), placed in exon 6. This variant was found in six patients, in all cases in compound heterozygosity with other *PIGQ* variants. In the two new cases, we identified these variants: in P1, a novel frameshift likely pathogenic (PVS1+PM2) variant c.1092dupC, p.(Phe365LeufsTer78) (Supplementary Figure S4). The Sanger sequencing chromatograms confirm the *PIGQ* gene variants in Patient 1 (P1). In P2, we found a missense variant of uncertain significance (VUS) (PM2) c.1370T>G, p.(Leu457Arg) (Supplementary Figure S5. The Sanger sequencing chromatograms confirm the *PIGQ* gene variants in Patient 2 (P2), in both cases in compound heterozygosity with in-frame deletion c.1199_1201del, p.(Tyr400del). The pathogenicity of the missense variant c.1370T>G, p.(Leu457Arg), that is, L457R, previously reported as a VUS, was proved by a functional study on the CHO (see below), and we changed its classification to likely pathogenic (PM2+PP3+PS3).

**TABLE 1:** Summary of the variants identified in the PIGQ-CDG cohort (NM_004204.3).

Patient	Variant in *PIGQ* gene	Exon/intron	Variant type	ACMG criteria (Richards et al., 2015)	Ethnicity	References
**P1**	**c.1199_1201del** **p.(Tyr400del)**	Exon 6	In-frame	Pathogenic (PP5+PM4+PM2)	Austrian	This study
	c.1092dupC p.(Phe365LeufsTer78)	Exon 6	Frameshift	Likely pathogenic (PVS1+PM2)		
**P2**	**c.1199_1201del p.(Tyr400del)**	Exon 6	In-frame	Pathogenic (PP5+PM4+PM2)	Austrian	This study
	c.1370T>G p.(Leu457Arg)	Exon 8	Missense	Likely pathogenic (PM2+PP3 +PS3)		
**P3 (St1)**	Homozygous c.1673del p.(Gly558AlafsTer65)	Exon 11	Nonsense	VUS (PM2)	Turkish	Johnstone et al. (2020)
**P4 (St2)**	**c.1199_1201del** **p.(Tyr400del)**	Exon 6	In-frame	Pathogenic (PP5+PM4+PM2)	European/Puerto Rico	Johnstone et al. (2020)
	c.942 + 1G>A IVS4+1G>A	Intron 4	Noncoding	Pathogenic (PVS1+PP5+PM2)		
**P5 (St3a)**	c.1640_1641del p.(Pro547GlnfsTer235)	Exon 11	Frameshift	Likely pathogenic (PVS1+PM2)	British Isles/French Canadian	Johnstone et al. (2020)
	**c.1199_1201del** **p.(Tyr400del)**	Exon 6	In-frame	Pathogenic (PP5+PM4+PM2)		
**P6 (St3b) brother**	c.1640_1641del p.(Pro547GlnfsTer235)	Exon 10	Frameshift	Likely pathogenic (PVS1+PM2)	British Isles/French Canadian	Johnstone et al. (2020)
	**c.1199_1201del** **p.(Tyr400del)**	Exon 6	In-frame	Pathogenic (PP5+PM4+PM2)		
**P7 (St4)**	c.1130_1168del p.(Ala377_Ser389del)	Exon 6	In-frame deletion	VUS (PM4, PM2)	Lebanese/Iraqi	Johnstone et al. (2020)
	c.1345G>C p.(Gly449Arg)	Exon 8	Missense	Likely pathogenic (PM3+PM2+PP3)		
**P8 (St5)**	c.49G>A p.(Gly17Arg)	Exon 2	Missense	VUS (PM2+PP3)	Mexican	Johnstone et al. (2020)
	c.942 + 1G>A IVS4+1G>A	Intron 4	Noncoding	Pathogenic (PVS1+PP5+PM2)		
**P9 (St6)**	Homozygous c.1732del p.(Asp578ThrfsTer45)	Exon 11	Frameshift	Likely pathogenic (PVS1+PM2)	Afghani	Johnstone et al. (2020)
**P10**	Homozygous c.690–2A>G	Intron 2	Noncoding	Pathogenic (PM3+PVS1+PM2)	West African	Martin et al. (2014)
**P11**	Homozygous: c.619C>T p.(Arg207Ter)	Exon 2	Nonsense	Pathogenic (PM3+PVS1+PM2)	n.a.	Alazami et al. (2015)
**P12**	c.968_969del p.(Leu323ProfsTer119)	Exon 5	Frameshift	Pathogenic (PM3+PVS1+PM2)	n.a.	Starr et al. (2019)
	**c.1199_1201del** **p.(Tyr400del)**	Exon 6	In-frame	Pathogenic (PP5+PM4+PM2)		
**P13**	Homozygous c.1631dupA p.(Tyr544fsTer79)	Exon 11	Nonsense	Likely pathogenic (PVS1+PM2)	Italian	Zanni et al. (2022)

Abbreviations: P, patient; St, abbreviation of each subject from original work (Johnstone et al., 2020); PP, pathogenicity supporting; PM, pathogenicity moderate; PVS, pathogenicity very strong (Richards et al., 2015); n.a., not available. Bold values represents the recurrent variant.

### 3.2 Pathogenicity validation of the missense variant L457R in the *PIGQ* gene in P2

**FIGURE 2:**
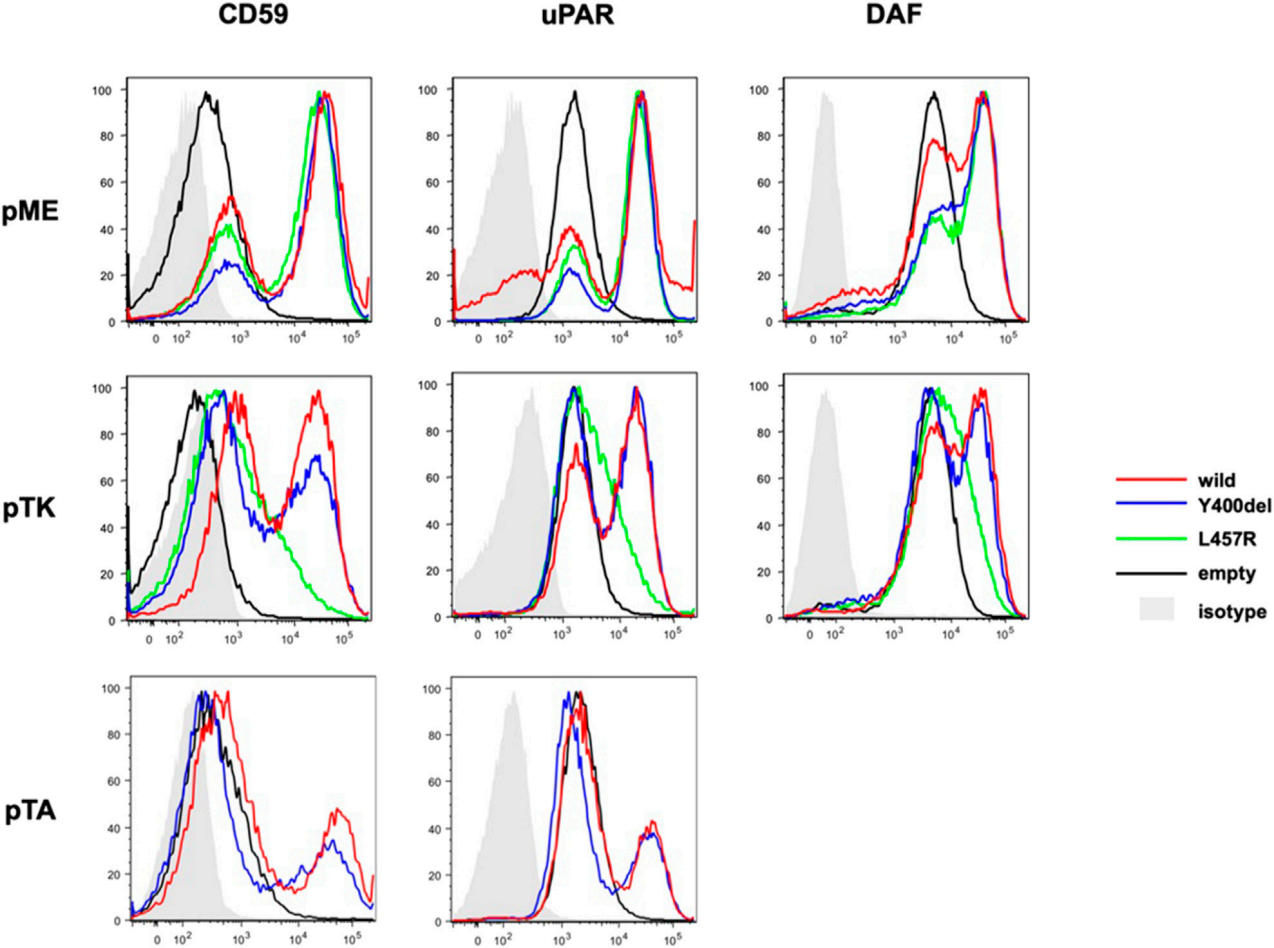
Pathogenicity validation of the variant L457R. Flow cytometry showed the restoration of the surface expression of GPI-APs in both mutants (Y400del mutant or L457R mutant PIGQ). The activity of Y400del mutant PIGQ appeared to be slightly reduced. L457R mutant PIGQ only partially restores the expression of GPI-APs, suggesting that L457R mutation is pathogenic (more detail in the main text).

Both mutants (Y400del and L457R) completely restored the surface expression of GPI-anchored proteins (GPI-APs). L457R mutant PIGQ, driven by a weak promoter (pTK), only partially restores the expression of GPI-APs, suggesting that the L457R mutation is pathogenic. The Y400del mutant driven by a weak promoter (pTK) or a minimum promoter (pTA) restored the expression of GPI-APs less efficiently than wild-type PIGQ, suggesting that the activity of Y400del mutant PIGQ was mildly decreased (Figure 2). The protein expression of the L457R mutant was drastically decreased, whereas the Y400del mutant was expressed at a similar level as the wild-type PIGQ (Supplementary Figure S3). The expression of CD109 and uPAR was slightly decreased in the patient’s fibroblasts (70% for uPAR, 32% for CD109, compared to the control), which was restored to the control level by retroviral transduction of wild-type PIGQ cDNA, suggesting that the reduction is due to PIGQ deficiency (Supplementary Figure S2).

### 3.3 Phenotype analysis and comparison

#### 3.3.1 Novel cases (P1 and P2)

**FIGURE 3:**
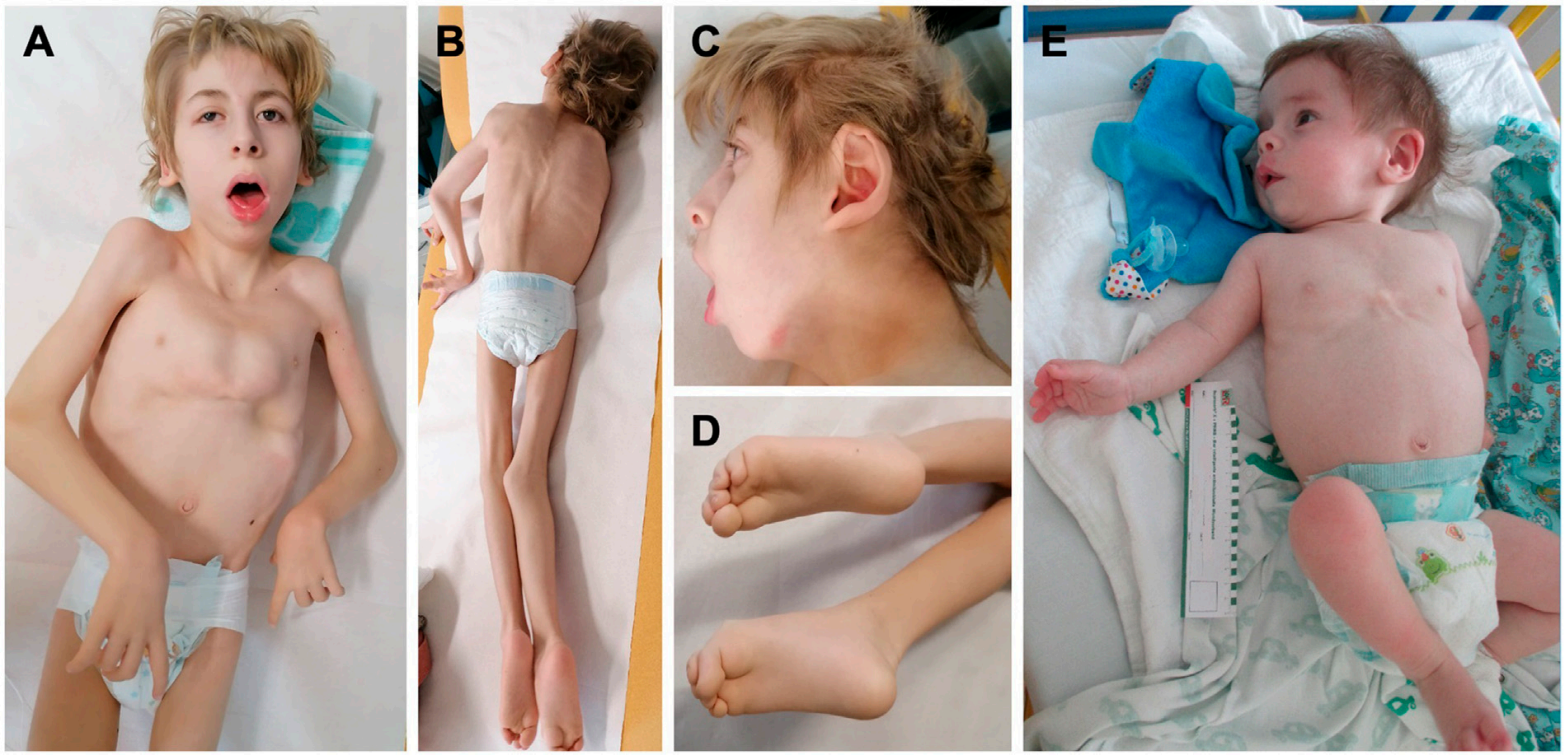
Phenotype of the two novel *PIGQ*-deficient patients. **(A)** P1 at the age of 12 years with coarse facial features, high arched eyebrow, downslanted palpebral fissures, anteverted nares, open mouth, pectus carinatum, generalized hypotrophy, contractures on extremities; **(B)** scoliosis; **(C)** dysmorphic earlobes; **(D)** crossing of the toes and their flexion contractures; **(E)** P2 at the age of 8 months, low-set ears with large ear lobes, depressed nasal bridge, anteverted nares, open mouth appearance, and pectus carinatum.

**FIGURE 4:**
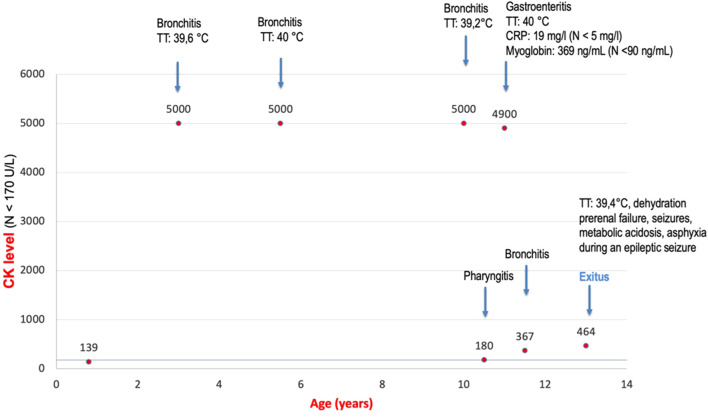
Attacks of rhabdomyolysis triggered by an infection in P1. The graph shows enzyme creatine kinase (CK) levels in serum in P1. At the first CK collection, the level was in the normal range. At this time, P1 did not have an infection. With every infection, levels increase to pathological levels.

Both novel patients (P1 and P2) were male subjects. Polyhydramnios as a prenatal complication was present in both patients, and P2 had also increased nuchal translucency (NT = 3.4 cm). The neonatal period was complicated in P1 by respiratory insufficiency, hypotonia, and hepatosplenomegaly and in P2 by neonatal icterus, hepatosplenomegaly, and severe global hypotonia (floppy infant). Both had craniofacial dysmorphism: P1 had coarse facial features, anteverted nares, open mouth, and macroglossia (Figures 3A–D), and P2 had large low-set ears with large ear lobes, depressed nasal bridge, anteverted nares, open mouth, and gingival enlargement (Figure 3E). Psychomotor milestones in P1 and P2 were delayed from the first months of life and worsened by age. Epilepsy in P1 started at age 3 months (infantile onset) and presented as infantile spasms with lifelong combined antiseizure medication needed to partially control the seizures. P2 also developed seizures during the first year of life and needed antiseizure medication for seizure control. Abnormal movements were present in both. Ocular presentation in P1 was poor eye contact, lagophthalmos, and nystagmus; in P2, visual fixation was lacking. The most visible skeletal changes in P1 were scoliosis and pectus carinatum (Figures 3A–D) and in P2 pectus carinatum (Figure 3E). P2 had no dentition until death. P2 had an insufficiency of the aortic valve, and prophylactic antibiotic treatment was needed. P2 had to be fed via a nasogastric tube because of feeding difficulties. In P1, an MRI of the brain showed significant enlargement of the bifrontal subarachnoid space, severe myelination delay, and decreased volume of cerebral white matter at 8 months of age. In P2, a brain MRI, including spectroscopy, showed changes compatible with hepatic encephalopathy or a previous hypoxic event and an enlargement of the left ventricle at 3 months of life. Alkaline phosphatase (ALP) was measured only in P1 with an elevated level of 632 U/l (N: 82–383). HyperCKemia was found only in P1, with creatine kinase (CK) levels higher than 5000 U/l (N: <170) during episodes of rhabdomyolysis. Episodes were triggered by an infection (primarily respiratory infections with elevated body temperature or fever) with a normal CK level at the beginning, in a noninfectious state (see Figure 4). Both patients died prematurely: P1 at the age of 13 years due to asphyxia during sepsis and P2 at 1 year of life due to pneumonia. More detailed phenotypes with a chronology of P1 and P2 signs are described in Supplementary materials.

#### 3.3.2 Phenotype summary of the MCAHS4 patient cohort

We summarized phenotypic data from 13 genetically confirmed *PIGQ*-deficient patients (n = 13; 11 already published and 2 novel); seven were male subjects (of which two were siblings), and five were female subjects. The median survival age of the patients was 5.5 years. The most common phenotypic features in all patients (i.e., 100%) in the studied group were hypotonia, psychomotor delay, epilepsy, MRI changes of the brain tissue, ocular abnormalities, and facial dysmorphism. A smaller proportion, that is, 90%, had abnormal movements and gastrointestinal issues. Less common were skeletal anomalies, issues in the neonatal period, genitourinary, teeth, and cardiac anomalies, elevation of ALP (in 62%), and prenatal complications in less than half of patients. The mentioned features are summarized in Table 2.

**TABLE 2:** Clinical manifestations of *PIGQ*-deficient patients (n = 13).

Features	P1	P2	All reported PIGQ patients together	%
Gender	M	M	5F, 7M (1 n.a.)	41/59
Prenatal complications	+	+	5/12 (1 n.a.)	41
Neonatal complications	+	+	9/12 (1 n.a.)	75
Hypotonia	+	+	**11/11 (2 n.a.)**	**100**
Developmental delay	+	+	**12/12 (1 n.a.)**	**100**
Epilepsy	+	+	**11/11 (2 n.a.)**	**100**
Neonatal onset (0–30 days)	-	-	1	
Infantile onset (1–12 months)	+	+	9	
Childhood onset (>1 year)	-	-	1	
Abnormal movements	+	+	10/11 (2 n.a.)	90
Facial dysmorphism	+	+	**12/12 (1 n.a.)**	**100**
Cardiac	+	-	8/12 (1 n.a.)	66
Genitourinary	-	-	7/11 (1 n.a.)	63
Ocular	+	+	**12/12 (1 n.a.)**	**100**
Skeletal	+	+	9/12 (1 n.a.)	75
Teeth	+	+	5/10 (3 n.a.)	50
GIT issues	-	+	9/10 (3 n.a.)	90
MRI findings	+	+	**9/9 (4 n.a.)**	**100**
Increased serum ALP	+	n.a.	5/8 (5 n.a.)	62
Ataxia	-	-	1 (12 n.a.)	
HyperCKemia/rhabdomyolysis	+	-	1/2 (11 n.a.)	50
Premature death	13 years	1 year	5.5 years	n.a.

Abbreviations: P, patient (new case); M, male; F, female; n.a., not available; y–, year/years.

### 3.4 GestaltMatcher analysis

#### 3.4.1 Facial similarity of MCAHS4 patients

**FIGURE 5:**
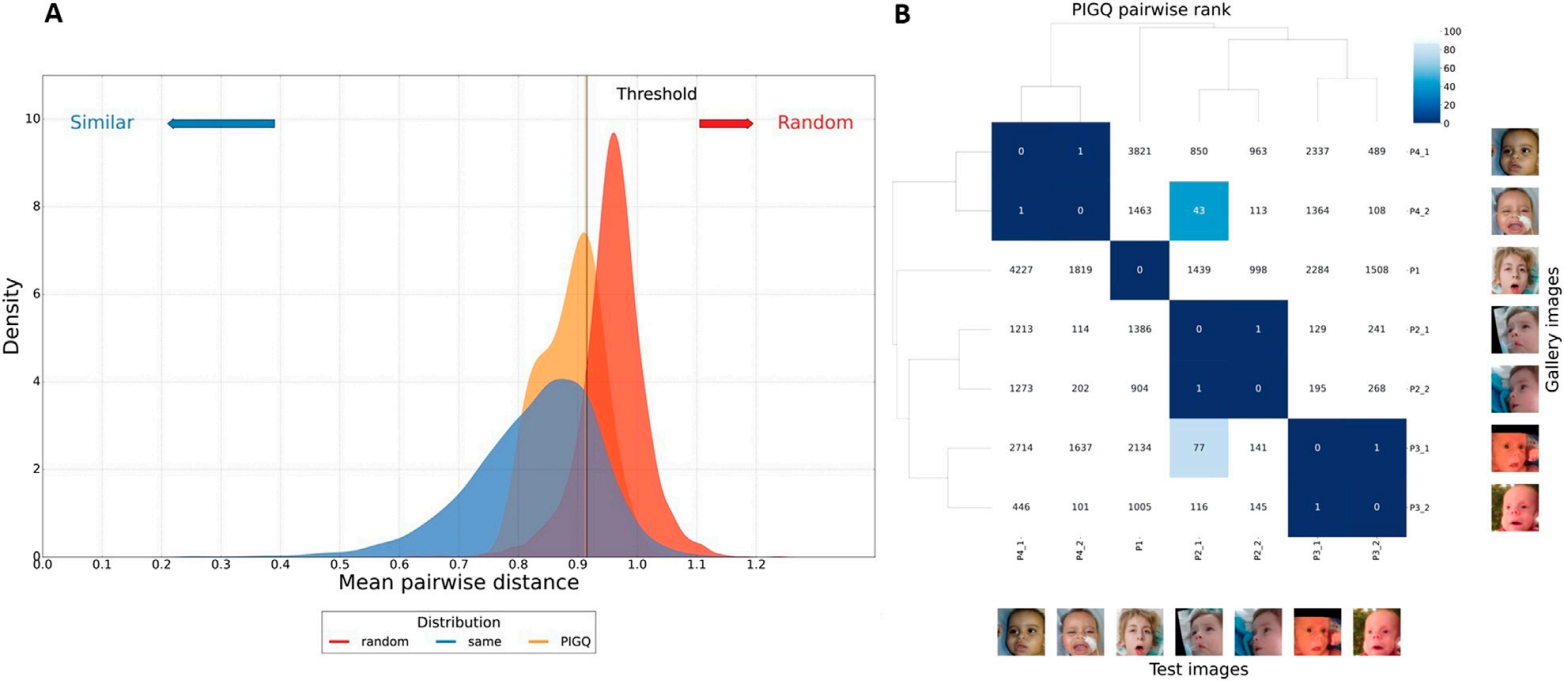
**(A)** Distance distribution comparison among MCAHS4 to the controls. The distance distribution among MCAHS4 individuals is depicted in orange, the random selection from the subjects with 328 disorders is shown in red, and the selection with the same disorder is illustrated in blue. The black vertical line is the threshold that classifies whether it is the same disorder or random selection. On the graph, 63.6% of the *PIGQ* distribution is below the threshold, indicating MCAHS4 individuals presenting. **(B)** GestaltMatcher pairwise rank analysis. The pairwise rank matrix displays the matched rank within the gallery of 7,459 images representing 449 syndromes. A smaller rank value (dark blue) indicates a higher similarity compared to other patients. For instance, consider column P2_1: Patient P4_2 is ranked 43rd (i.e., the 43rd most similar patient) among the 7,459 images representing other syndromes.

Finally, we collected seven photos from four *PIGQ*-deficient subjects (n = 4). We first calculated PIGQ individuals’ mean pairwise distance and random sampled 100 times to validate their similarities. We made sure the images from the individual would not be sampled together to avoid bias. In Figure 5A, we compare the PIGQ distribution (orange) to two distributions (identical and random) built from the 1,555 images from different subjects with 328 syndromes from the GestaltMatcher Database (GMDB) (Lesmann et al., 2024). For each of the 328 syndromes, we randomly selected a sub-cohort and computed the mean pairwise distance 100 times to build the “same” distribution (shown in blue). Additionally, we generated the “random” distribution (shown in red) by randomly sampling a sub-cohort without constraining them within the same syndrome and calculating their mean pairwise distance 100 times.

Our five-fold cross-validation with receiver operating characteristic (ROC) analysis yielded a significant threshold for distinguishing the identical and random distributions. The threshold c was determined by the maximum Youden index, resulting in c = 0.909, corresponding to a sensitivity of 0.862 and a specificity of 0.792. Notably, 63.6% of the PIGQ distribution was below the threshold, indicating the cohort was similar.

#### 3.4.2 GestaltMatcher pairwise rank analysis

We further utilized pairwise comparison analysis to investigate the facial similarities at the individual level. We compared seven images of four individuals with PIGQ to 7,459 images with 449 different disorders from GMDB by performing the leave-one-out cross-validation to simulate the real-world scenario. We can visualize the similarity of each pair of individuals compared to the control cohort. Figure 5B shows the results of testing the image of P2_1, adding the remaining six images in the space with the other 7,459 pictures, and calculating the ranks of these images to P2_1. It shows that image P4_2 was at the 43rd closest position to P2_1.

We also examined the distribution of the most frequently matched syndromes across the top-30 closest images for each PIGQ individual, as summarized in Table 1. The results show that MCAHS was the most frequently predicted disorder group among the top-ranked matches. Although PIGQ is clinically associated with the MCAHS group, it does not appear in Table 1 because none of the PIGQ images ranked within the top-30 matches of each other—the best match was ranked 43rd. This highlights both a limitation of the current reference database and the challenge of matching ultra-rare conditions. Nevertheless, the frequent retrieval of MCAHS-related cases suggests that GestaltMatcher may still guide clinicians toward the correct phenotypic neighborhood, thereby narrowing the diagnostic search space even when the precise disorder is not yet represented in the database.

## 4 Discussion

Our work presents two novel cases of MCAHS4 patients with two novel variants in the *PIGQ* gene. In both cases, the variant was found as compound heterozygosity with in-frame deletion c.1199_1201del, p.(Tyr400del), which is the most common disease-causing variant found in the *PIGQ* gene. The first novel variant, c.1092dupC, p.(Phe365LeufsTer78), found in P1, is frameshift, according to ACMG recommendation evaluated as likely pathogenic (PVS1+PM2) (Richards et al., 2015). In this case, we considered the ACMG classification sufficient to assess the pathogenicity of the detected *PIGQ* variant. Unfortunately, we did not have the necessary material available for possible functional studies. The pathogenicity of the missense variant c.1370T>G, p.(Leu457Arg), previously evaluated as a VUS, found in P2, was proven by a functional study on CHO (according to the protocol published by Martin et al., 2014). When PIGQ-deficient CHO cells were transfected with wild-type or mutant *PIGQ* cDNA driven by a strong promoter (pME), both the L457R and Y400del mutants restored the surface expression of GPI-APs to a level similar to that of wild-type *PIGQ*. However, protein expression was significantly reduced, suggesting that the L457R mutant protein is unstable. When CHO cells were transfected with wild-type and mutant PIGQ cDNA driven by a weaker promoter (pTK or pTA), restoration of GPI-APs by the L457R mutant was significantly decreased (pTK), while that by the Y400del mutant was only mildly reduced (pTK or pTA). Although this functional assay is based on an artificial system and may not fully reflect the *in vivo* situation, it allows for the comparison of the activity between wild-type and mutant constructs. Currently, it is not possible to establish genotype–phenotype correlations due to limited phenotype data and small sample size.

Phenotypic features present in all (100%) MCAHS4 individuals are generalized hypotonia, global developmental delay, epilepsy, visual impairment, and interindividual variable brain MRI changes in concordance with data already published by Johnstone et al., 2020. Alkaline phosphatase (ALP) is elevated only in 62% of cases. Nevertheless, the association of the above-mentioned features with elevated ALP levels is highly suggestive of a disease from the group of disorders of glycosylphosphatidylinositol biosynthesis, including PIGQ-CDG, that is, MCAHS4 (Carmody et al., 2020; Ng et al., 2024).

According to the already published data, all MCAHS4 patients (100%) present with craniofacial dysmorphism, including coarse facial features, macroglossia, and abnormalities in cranial shape (Johnstone et al., 2020). In both novel patients (P1 and P2), in addition to other published MCAHS4 patient photographs, we spotted craniofacial dysmorphism, which seems to be similar. The traits that seem to be shared in all cases are Coarse facial features HP:0000280 and anteverted nares HP:0000463. According to the Human Phenotype Ontology (Gargano et al., 2024), these terms are associated with the *PIGQ* gene. However, the *PIGQ* gene is not listed in the case of the trait Open mouth, HP:0000194, but based on our knowledge, this sign is present in all MCAHS4 patients and should be considered a common trait in MCAHS4 patients. GestaltMatcher analysis shows differences between cohort and controls. These findings suggest that computational facial analysis may be helpful in guiding the diagnosis of potential MCAHS4 patients by identifying phenotypic similarity. However, given that the current analysis is based on only four individuals, the results should be interpreted with caution. The observed clustering may be influenced by the small sample size and limited diversity within the cohort. Future studies with larger, more diverse patient groups are necessary to validate and refine these findings.

Rhabdomyolysis or hyperCKemia has not been described in MCAHS4 patients until now. P1 had attacks of rhabdomyolysis induced by a febrile infection, with very high levels of creatine kinase (CK) in the blood serum (CK > 5000 U/l). Bioinformatic reanalysis of the WES data has been done in P1, but no other genetic cause (except variants in the *PIGQ* gene) has been found to be responsible for rhabdomyolysis. Reviewing the literature with a focus on the other genes involved in the GPI-biosynthesis pathway (Synthesis Group) (Carmody et al., 2020), we found that the *DPM2* gene (OMIM: * 603564) related to autosomal recessive congenital disorder of glycosylation, type Iu (OMIM: # 615042), that is, DPM2-CDG (Ng et al., 2024), is associated with hyperCKemia. The *DPM2* gene encodes dolichol-phosphate mannosyltransferase-2, the enzyme responsible for adequate glycosylation of muscular proteins in the endoplasmic reticulum. These glycosylated proteins, for example, sarcoglycans and dystroglycans, are needed for muscle cell integrity. In case of inadequate glycosylation, myocytes are unstable, damaged, and release myocytes intracellular content, including CK, myoglobin, etc., to the blood (Radenkovics et al., 2021; Zhao et al., 2023). Another gene involved in GPI-anchor biosynthesis, responsible for hyperCKemia is the *PIGY* gene (OMIM: * 610662), associated with hyperphosphatasia with impaired intellectual development syndrome 6 (OMIM: # 616809), that is, PIGY-CDG. Ilkovski et al., 2015 described two siblings in whom creatine kinase was persistently elevated in both siblings (554–3640 U/l, N: 15–180 U/l) (Ilkovski et al., 2015). *PIGQ*, and also *PIGY* and *DPM2*, are genes from the GPI-anchor Synthesis Group (Carmody et al., 2020) involved in the first step of the GPI-anchor biosynthesis. We assume that the damaged anchoring process of myocytes caused by insufficiency of the *PIGQ, PIGY*, and *DPM2* genes leads to rhabdomyolysis and hyperCKemia. It remains unclear why we could see attacks of rhabdomyolysis triggered by an infection in our patient (P1) while *PIGY* and *DPM2* patients have consistently elevated levels of CK. It is possible that hyperCKemia is also associated with other GPI-anchor biosynthesis genes (Synthesis group as well as Transamidase + Remodeling Group), based on muscular vulnerability due to GPI-anchor dysfunction. The presence and detailed pathomechanism of rhabdomyolysis in these genes should be investigated more thoroughly in the future. However, it is appropriate to consider attacks of rhabdomyolysis in MCAHS4 patients because of their potential life-threatening complications. As a limitation of our study, we see a small sample size in deep gestalt analysis by GestalMatcher and limited clinical data, including hyperCKemia and rhabdomyolysis, in the published data of an actual group of 13 MCAHS4 patients. Therefore, we consider our conclusions as a hypothesis-generating finding that require further validation.

## Data Availability

The datasets presented in this study can be found in online repositories. The names of the repository/repositories and accession number(s) can be found in the article/Supplementary Material.

## References

[B1] Alazami A. M. Patel N. Shamseldin H. E. Anazi S. Al-Dosari M. S. Alzahrani F. (2015). Accelerating novel candidate gene discovery in neurogenetic disorders via whole-exome sequencing of prescreened multiplex consanguineous families. Cell Rep. 10 (2), 148–161. 10.1016/j.celrep.2014.12.015 25558065

[B2] Carmody L. C. Blau H. Danis D. Zhang X. A. Gourdine J. P. Vasilevsky N. (2020). Significantly different clinical phenotypes associated with mutations in synthesis and transamidase+remodeling glycosylphosphatidylinositol (GPI)-anchor biosynthesis genes. OJRD 15 (1), 40. 10.1186/s13023-020-1313-0 32019583 PMC7001271

[B3] Gargano M. A. Matentzoglu N. Coleman B. Addo-Lartey E. B. Anagnostopoulos A. V. Anderton J. (2024). The Human Phenotype Ontology in 2024: phenotypes around the world. Nucl. Acids Res. 52 (D1), D1333–D1346. 10.1093/nar/gkad1005 37953324 PMC10767975

[B4] Hamosh A. Scott A. F. Amberger J. S. Bocchini C. A. McKusick V. A. (2005). Online Mendelian Inheritance in Man (OMIM), a knowledgebase of human genes and genetic disorders. Nucl. Acids Res. 33 (Database issue), D514–D517. 10.1093/nar/gki033 15608251 PMC539987

[B5] Hsieh T. C. Bar-Haim A. Moosa S. Ehmke N. Gripp K. W. Pantel J. T. (2022). GestaltMatcher facilitates rare disease matching using facial phenotype descriptors. Nat. Genet. 54 (3), 349–357. 10.1038/s41588-021-01010-x 35145301 PMC9272356

[B6] Hustinx A. Hellmann F. Sümer Ö. Javanmardi B. André E. Krawitz P. (2023). “Improving deep facial phenotyping for ultra-rare disorder verification using model ensembles,” in IEEE/CVF winter conference on applications of computer vision (WACV) (IEEE, 2023). 10.1109/WACV56688.2023.00499

[B7] Ilkovski B. Pagnamenta A. T. O'Grady G. L. Kinoshita T. Howard M. F. Lek M. (2015). Mutations in PIGY: expanding the phenotype of inherited glycosylphosphatidylinositol deficiencies. Hum. Mol. Genet. 24 (21), 6146–6159. 10.1093/hmg/ddv331 26293662 PMC4599673

[B8] Johnstone D. L. Nguyen T. T. M. Zambonin J. Kernohan K. D. St-Denis A. Baratang N. V. (2020). Early infantile epileptic encephalopathy due to biallelic pathogenic variants in PIGQ: report of seven new subjects and review of the literature. JIMD 43 (6), 1321–1332. 10.1002/jimd.12278 32588908 PMC7689772

[B9] Kinoshita T. (2014). Biosynthesis and deficiencies of glycosylphosphatidylinositol. Physic. Biol. Sci. 90 (4), 130–143. 10.2183/pjab.90.130 PMC4055706 24727937

[B10] Kinoshita T. (2020). Biosynthesis and biology of mammalian GPI-anchored proteins. Open Biol. 10 (3), 190290. 10.1098/rsob.190290 32156170 PMC7125958

[B11] Knaus A. Kortüm F. Kleefstra T. Stray-Pedersen A. Đukić D. Murakami Y. (2019). Mutations in PIGU impair the function of the GPI transamidase complex, causing severe intellectual disability, epilepsy, and brain anomalies. Am. J. Hum. Genet. 105 (2), 395–402. 10.1016/j.ajhg.2019.06.009 31353022 PMC6698879

[B12] Lesmann H. Hustinx A. Moosa S. Klinkhammer H. Marchi E. Caro P. (2024). GestaltMatcher Database - a global reference for facial phenotypic variability in rare human diseases. medRxiv Prepr. Available online at:. https://www.medrxiv.org/content/10.1101/2023.06.06.23290887v4 (Accessed April 26, 2025). 10.21203/rs.3.rs-4438861/v1

[B13] Martin H. C. Kim G. E. Pagnamenta A. T. Murakami Y. Carvill G. L. Meyer E. (2014). Clinical whole-genome sequencing in severe early-onset epilepsy reveals new genes and improves molecular diagnosis. Hum. Mol. Genet. 23 (12), 3200–3211. 10.1093/hmg/ddu030 24463883 PMC4030775

[B14] Ng B. G. Freeze H. H. Himmelreich N. Blau N. Ferreira C. R. (2024). Clinical and biochemical footprints of congenital disorders of glycosylation: proposed nosology. Mol. Genet. Met. 142 (1), 108476. 10.1016/j.ymgme.2024.108476 PMC11251693 38653092

[B15] Radenkovic S. Fitzpatrick-Schmidt T. Byeon S. K. Madugundu A. K. Saraswat M. Lichty A. (2021). Expanding the clinical and metabolic phenotype of DPM2 deficient congenital disorders of glycosylation. Mol. Genet. Met. 132 (1), 27–37. 10.1016/j.ymgme.2020.10.007 PMC7855207 33129689

[B16] Richards S. Aziz N. Bale S. Bick D. Das S. Gastier-Foster J. (2015). Standards and guidelines for the interpretation of sequence variants: a joint consensus recommendation of the American College of medical genetics and genomics and the association for molecular pathology. Genet. Med. official J. Am. Coll. Med. Genet. 17 (5), 405–424. 10.1038/gim.2015.30 PMC4544753 25741868

[B17] Starr L. J. Spranger J. W. Rao V. K. Lutz R. Yetman A. T. (2019). PIGQ glycosylphosphatidylinositol-anchored protein deficiency: characterizing the phenotype. Am. J. Med. Genet. Part A 179 (7), 1270–1275. 10.1002/ajmg.a.61185 31148362

[B18] Tiede A. Daniels R. J. Higgs D. R. Mehrein Y. Schmidt R. E. Schubert J. (2001). The human GPI1 gene is required for efficient glycosylphosphatidylinositol biosynthesis. Gene 271 (2), 247–254. 10.1016/s0378-1119(01)00510-8 11418246

[B19] Watanabe R. Inoue N. Westfall B. Taron C. H. Orlean P. Takeda J. (1998). The first step of glycosylphosphatidylinositol biosynthesis is mediated by a complex of PIG-A, PIG-H, PIG-C and GPI1. EMBO J 17 (4), 877–885. 10.1093/emboj/17.4.877 9463366 PMC1170437

[B20] Wu T. Yin F. Guang S. He F. Yang L. Peng J. (2020). The Glycosylphosphatidylinositol biosynthesis pathway in human diseases. Orphanet J. Rare Dis. 15, 129. 10.1186/s13023-020-01401-z 32466763 PMC7254680

[B21] Zanni G. D'Abrusco F. Nicita F. Cascioli S. Tosi M. Corrente F. (2022). PIGQ-related glycophosphatidylinositol deficiency associated with nonprogressive congenital ataxia. Cerebellum Lond. Engl. 21 (4), 525–530. 10.1007/s12311-021-01288-x 34089469

[B22] Zhao P. Hu Y. Hu J. Li C. Huang Y. Zhang L. (2023). Identification and characterization of a new variation in DPM2 gene in two Chinese siblings with mild intellectual impairment. Front. Genet. 14, 930692. 10.3389/fgene.2023.930692 37152991 PMC10154465

