# Supplementary figures and images for: Two novel cases with PIGQ-CDG: expansion of the genotype–phenotype spectrum and evaluation of GestaltMatcher as a diagnostic tool
Source: Front Genet. 2025 Jul 11;16:1598602. doi: 10.3389/fgene.2025.1598602 (PMC12289473; doi:10.3389/fgene.2025.1598602)

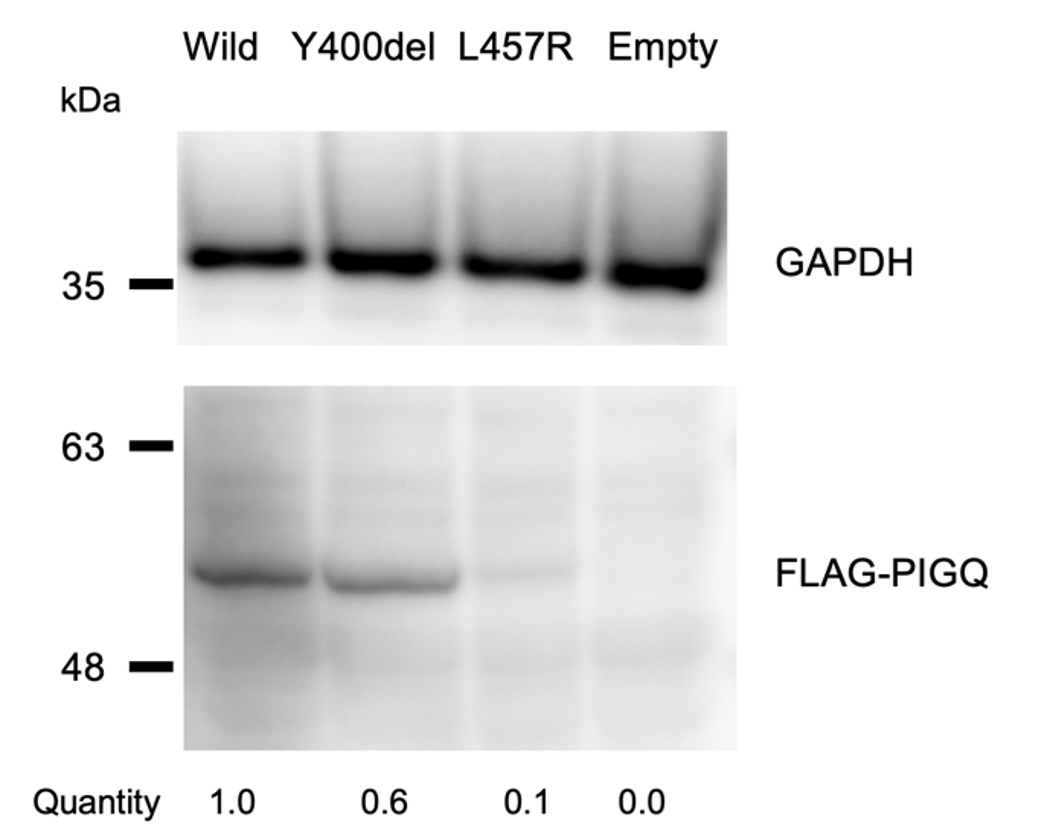

Supplement: Supplementary file 1 [file Image3.jpeg]

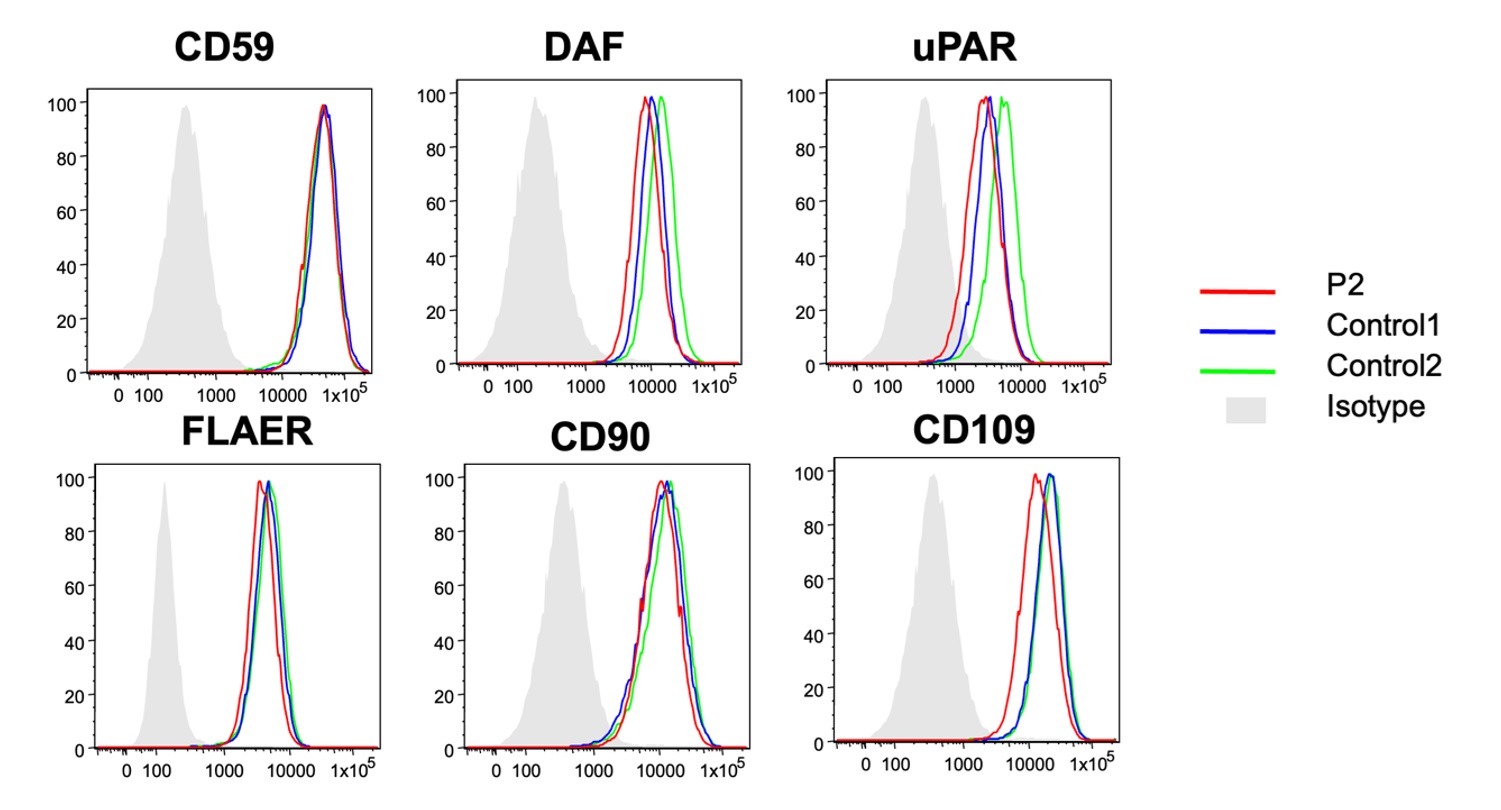

Supplement: Supplementary file 3 [file Image1.jpeg]

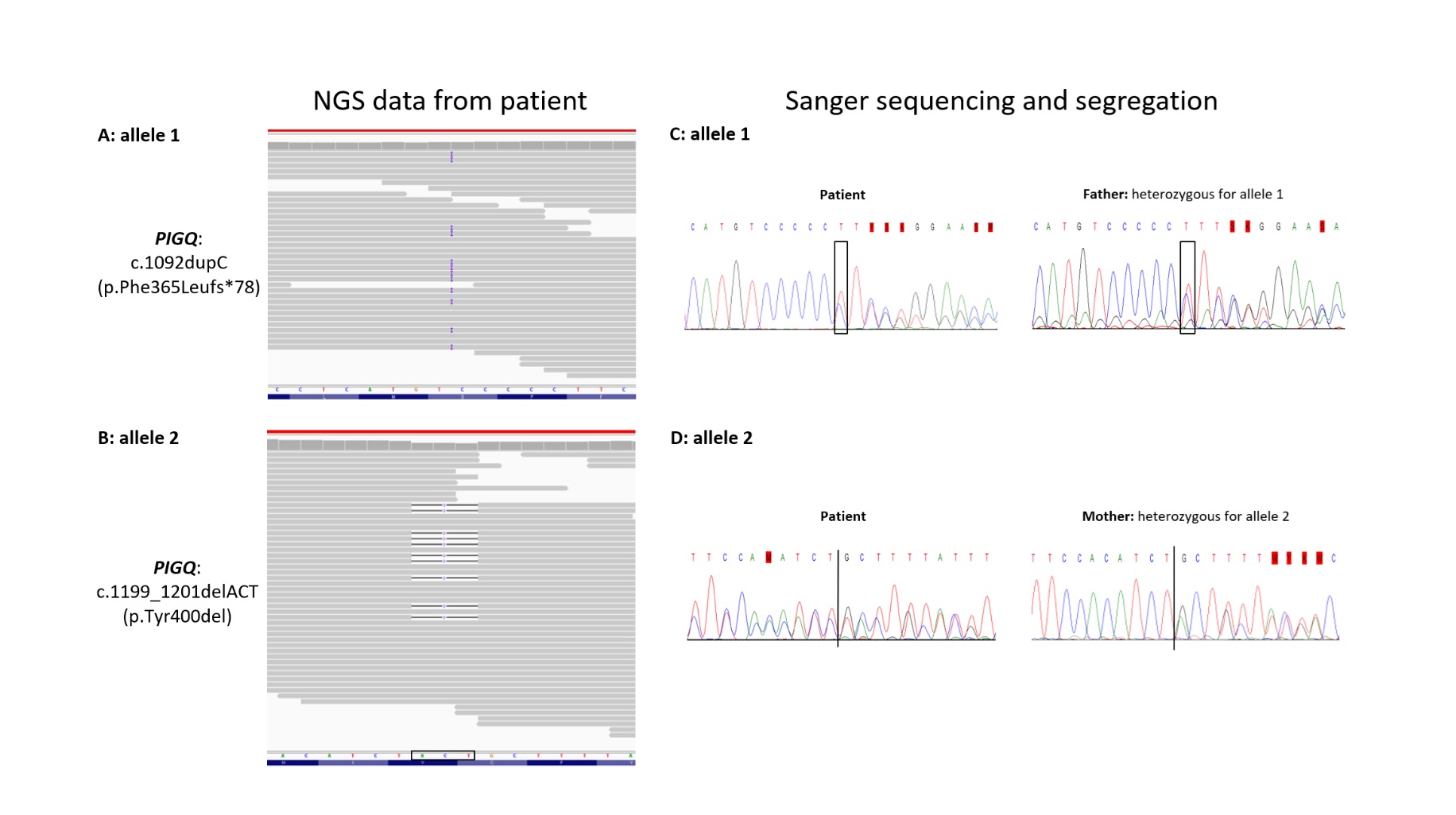

Supplement: Supplementary file 4 [file Image4.jpeg]

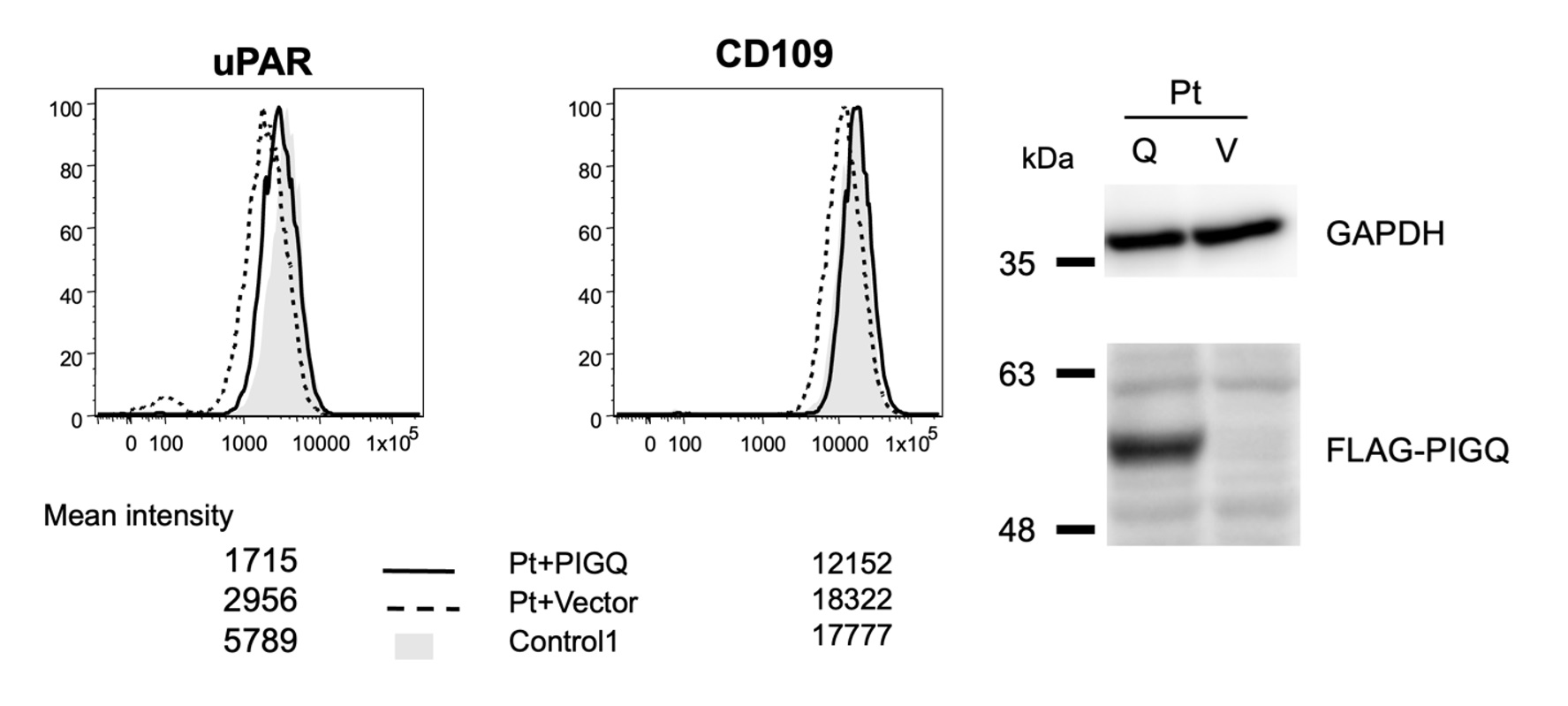

Supplement: Supplementary file 5 [file Image2.jpeg]

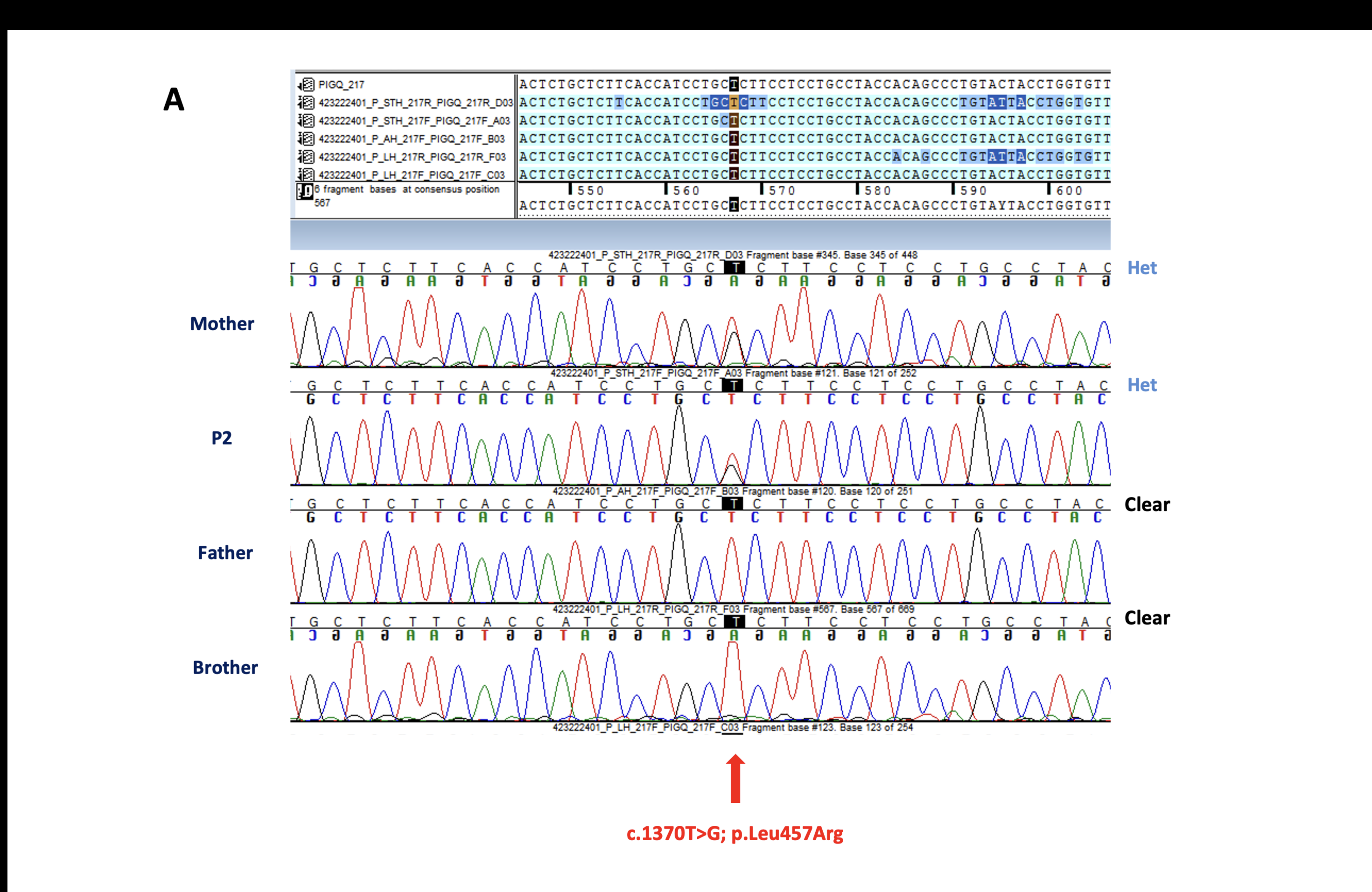

Supplement: Supplementary file 6 [file Image5.png]

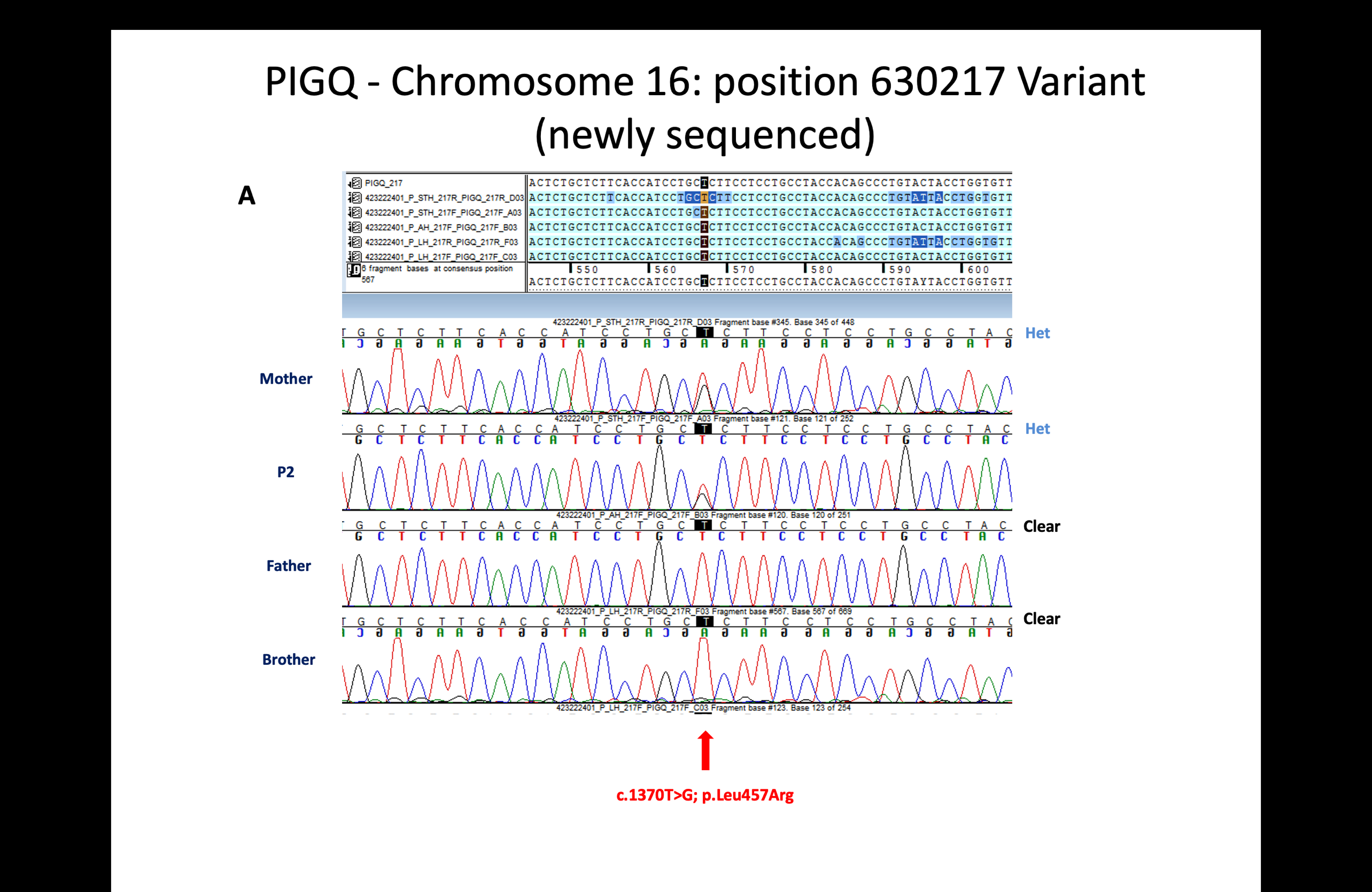

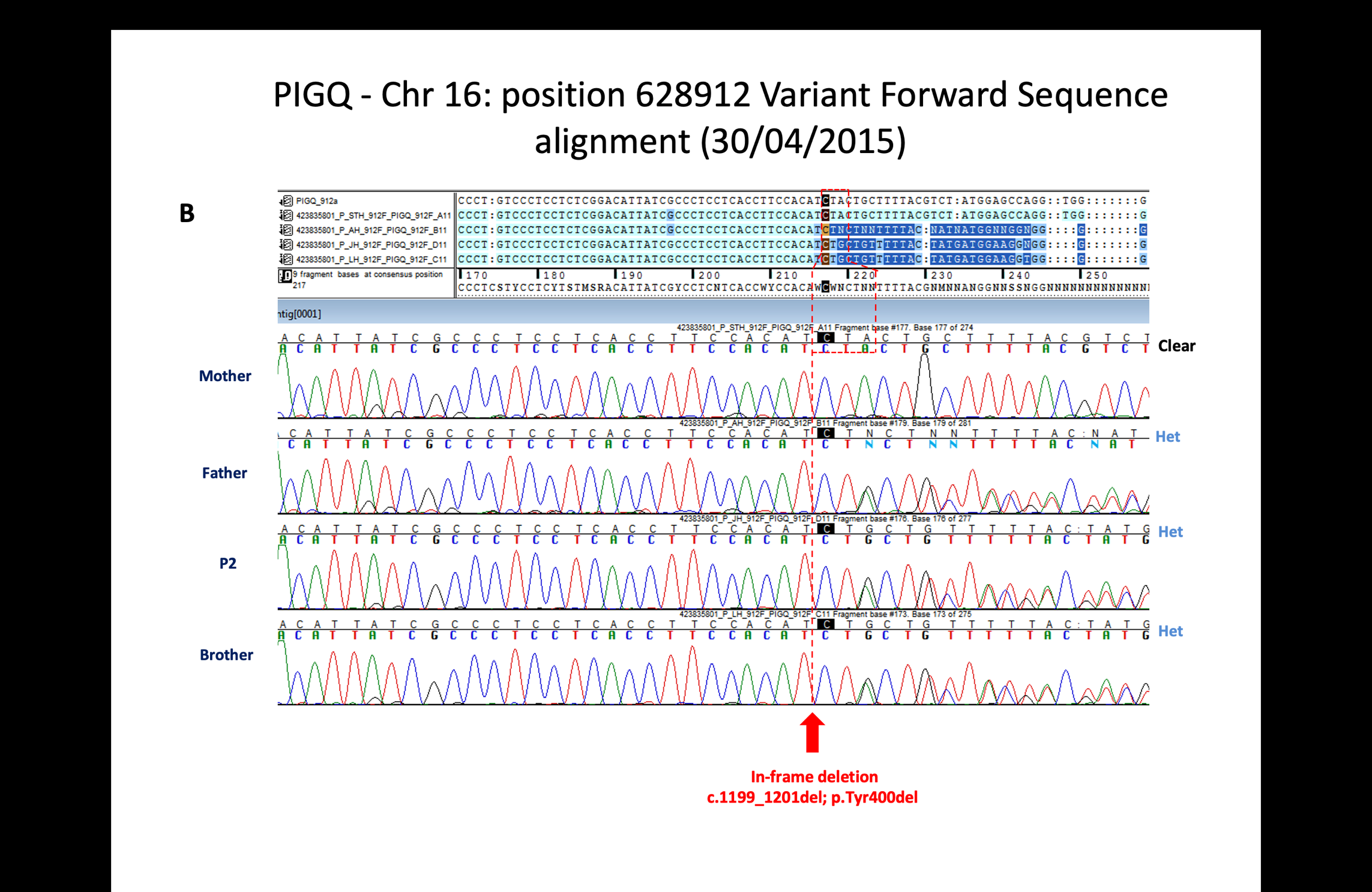

Supplement: Supplementary file 7 [file DataSheet2.docx]

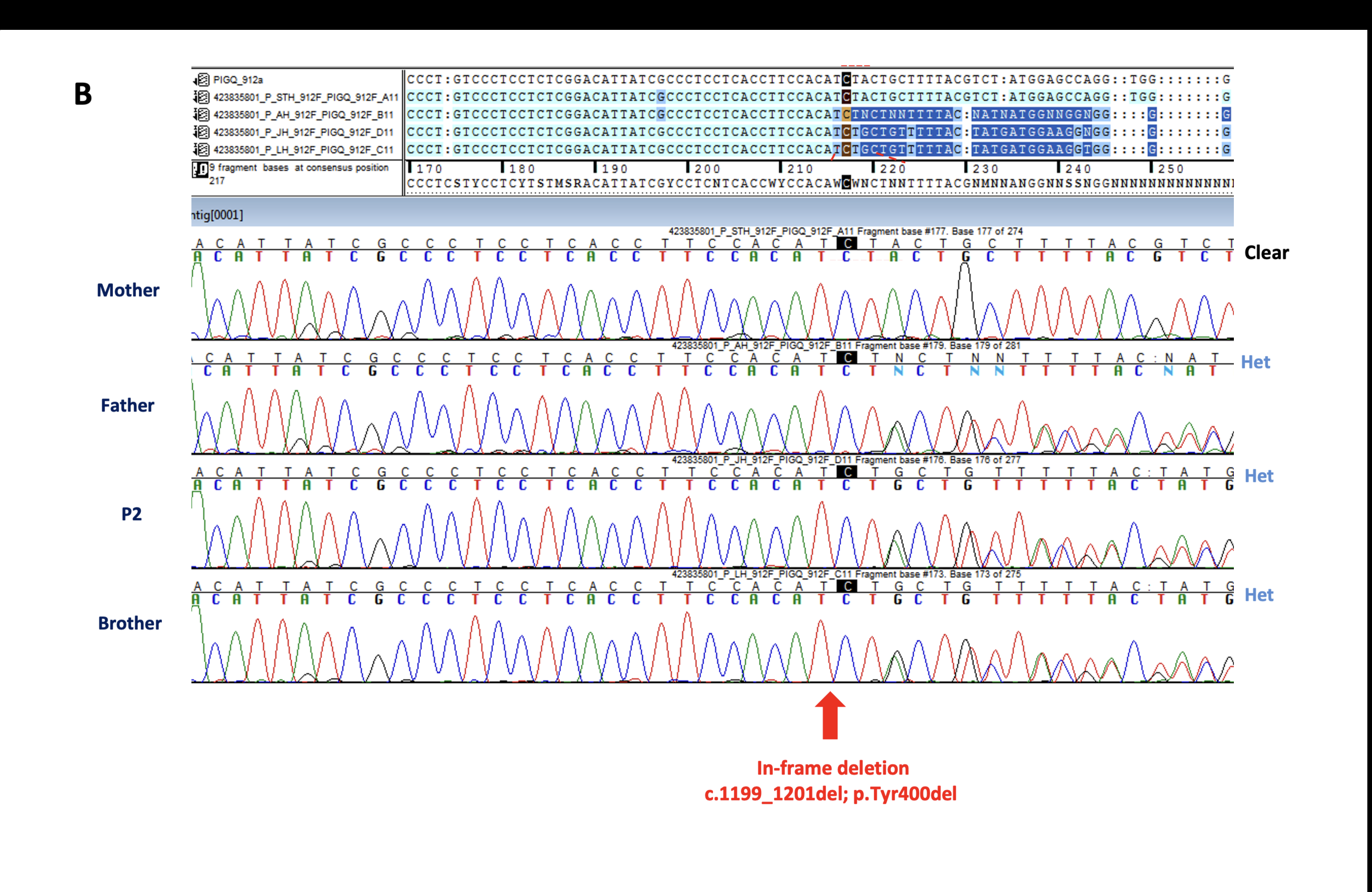

Supplement: Supplementary file 8 [file Image6.png]
