# Supplementary material for: Two novel cases with PIGQ-CDG: expansion of the genotype–phenotype spectrum and evaluation of GestaltMatcher as a diagnostic tool
Source: Front Genet. 2025 Jul 11;16:1598602. doi: 10.3389/fgene.2025.1598602 (PMC12289473; doi:10.3389/fgene.2025.1598602)
Supplement: Supplementary file 2 [file Table1.docx]

| **Patient** | **P1** | **P2** | **P3 (St1)** | **P4 (St2)** | **P5 (St3a)** | **P6 (St3b)** | **P7 (St4)** | **P8 (St5)** | **P9 (St6)** | **P10** | **P11** | **P12** | **P13** |
| --- | --- | --- | --- | --- | --- | --- | --- | --- | --- | --- | --- | --- | --- |
| **Reference** | This study | This study | Johnstone et al., 2020 | Johnstone et al., 2020 | Johnstone et al., 2020 | Johnstone et al., 2020 | Johnstone et al., 2020 | Johnstone et al., 2020 | Johnstone et al., 2020 | Martin et al., 2014 | Alazami et al., 2015 | Starr et al., 2019 | Zanni et al., 2021 |
| **Sex** | M | M | F | F | F | F | F | M | M | M | N/A | M | M |
| **Mutation** | c.1199_1201del  p.(Tyr400del)  c.1092dupC  p.(Phe365LeufsTer78) | c.1199_1201del p.(Y400del)  c.1370T>G  p.(Leu457Arg) | Homozygous  c.1673del  p.(Gly558AlafsTer65) | c.1199_1201del  p.(Tyr400del)  c.942+1G>A | c.1640_1641del  p.(Pro547GlnfsTer235)  c.1199_1201del  p.(Tyr400del) | c.1640_1641del  p.(Pro547GlnfsTer235)  c.1199_1201del  p.(Tyr400del) | c.1130_1168del  p.(Ala377_S389del)  c.1345G>C  p.(Gly449Ary) | c.49G>A  p.(Gly17Arg)  c.942+1G>A | Homozygous  c.1732del  p.(Asp578ThrfsTer45) | Homozygous  c.690-2A>G | Homozygous:  c.619C>T  p.(Arg207Ter) | c.968_969del  p.(Leu323ProfsTer119)  c.1199_1201del  p.(Tyr400del) | Homozygous c.1631dupA p.(Tyr544fsTer79) |
| **Ethnicity** | Austrian | Austrian | Turkish | European/Puerto Rican | British Isles/French Canadian | British Isles/French Canadian | Lebanese/Iraqi | Mexican | Afghani | West African | N/A | N/A | Italian |
| **Current age/age of death** | death  at 13 y | death  at 1 y | alive  at 11 y | alive  at 6y 6 m | death  at 2 d | death  at 5 y | death  at 9 m | alive  at 2 y 2 m | death  at 3 y 9 m | death  at 2 y 4 m | N/A | death  at 10 m | alive  at 10 y |
| **Polyhydramnios** | + | +  NT 3,4 | - | + | - | - | + | - | - | - | N/A | + | - |
| **Newborn period** | hsm | hsm |  |  |  |  |  |  |  |  |  |  |  |
| *Feeding difficulties* | - | + | - | - | - | - | - | + | + | - | N/A | + | - |
| *Respiratory distress* | + | - | - | + | + | - | + | + | - | - | N/A | - | - |
| *Jaundice* | - | + | - | - | - | + | - | + | - | - | N/A | - | - |
| **Neurologic** |  |  |  |  |  |  |  |  |  |  |  |  |  |
| *Hypotonia* | + | + | + | + | N/A | + | + | + | + | + | N/A | + | + |
| *Developmental delay* | + | + | + | + | N/A | + | + | + | + | + | + | + | + |
| *Epilepsy onset* | 3 m | < 1 y | 6 m | 7 m | N/A | 4 m | 7 m | 6 m | 2.5 m | 1 m  Ohtahara syndrome | N/A | 7 m | 8 y absence/d GTCS/y |
| *Abnormal movements* | + | + | + | + | N/A | + | + | + | + | + | N/A | + | ataxia |
| *Brain MRI changes* | enlargement of bifrontal subarachnoid space, severe myelination delay | enlargement of the left ventricle, normal myelinization of the dorsal internal capsule | broad periventricular space, poor myelination | prominent frontal horns of the lateral ventricles, periventricular leukomalacia | broad periventricular space, poor myelinization | volume loss of vermis and cortex, enlargement of lateral ventricle loss of subcortical white matter, poor myelinization | volume loss of vermis and cortex, poor myelination, dangling choroids, pituitary hypoplasia with preservation of the stalk | N/A | N/A | N/A | N/A | ventriculomegaly (left > right) | cerebellar atrophy |
| **Skelet/teeth** |  |  |  |  |  |  |  |  |  |  |  |  |  |
| *Teeth anomalies* | + | gingival enlargement | - | + | N/A | + | N/A | + | - | - | N/A | - | - |
| *Cranial shape anomalies* | - | - | - | + | - | - | + | + | + | - | N/A | + | N/A |
| *Skeletal anomalies* | + | +/- | + | + | - | + | + | + | - | - | N/A | + | scoliosis |
| *Serum ALP elevation* | + | N/A | - | + | N/A | + | + | N/A | - | N/A | N/A | + | - |
| *Join contractures* | + |  | + | - | - | + | + | - | - | - | N/A | N/A | - |
| **Ophtalmological anomalies** | + | + | + | + | N/A | + | + | + | + | + | + | + | + |
| **Dysmorphic features** | + | + | + | + | + | + | + | + | + | + | N/A | + | + |
| **Genitourinary anomalies** | - | - | + | + | + | N/A | + | + | + | - | N/A | + | - |
| **Others** |  |  |  |  |  |  |  |  |  |  |  |  |  |
| *Rhabdomyolysis* | + | - | N/A | N/A | N/A | N/A | N/A | N/A | N/A | N/A | N/A | N/A | N/A |
| *HyperCKemia* | + | - | N/A | N/A | N/A | N/A | N/A | N/A | N/A | N/A | N/A | N/A | N/A |
| **Cardiac anomalies** | - | - | - | + | + | + | + | + | + | - | N/A | + | - |
| **GIT issues** | - | +/- | + | + | N/A | + | + | + | + | + | N/A | + | N/A |

**Supplementary Table 1. Review of the literature – clinical features of the patients with biallelic pathogenic variants in the *PIGQ* gene**

**Abbreviations:** P – patient; M/F – male/female; y – year; m – month; d – day.; NT – nuchal translucency; hsm – hepatosplenomegaly; N/A – not available information, ALP – alkaline phosphatase; GTCS – generalized tonic-clonic seizures
