# Supplementary material for: Two novel cases with PIGQ-CDG: expansion of the genotype–phenotype spectrum and evaluation of GestaltMatcher as a diagnostic tool
Source: Front Genet. 2025 Jul 11;16:1598602. doi: 10.3389/fgene.2025.1598602 (PMC12289473; doi:10.3389/fgene.2025.1598602)

**Supplementary Material (Text part)**

**1 Genetic testing**

**1.1 DNA isolation**

**Patient 1 (P1):** Extraction of patient´s DNA from peripheral blood was performed according to standard protocols described in the manufacturer’s instructions using (Kit Promega Maxwell 16 Blood Kit or Maxwell RSC Whole Blood Kit).

**Patient 2 (P2):** DNA was extracted from peripheral blood leucocytes in EDTA using standard spin-column-based methods.

**1.2 DNA sequencing**

**P1:** Enrichment for whole-exome analysis was performed with the TruSeq Exome Kitfrom Illumina (San Diego, California, USA). DNA fragments were paired end sequenced on an Illumina NextSeq500 system. In patient 2 a multigene panel testing was enriched for 6713 genes associated with Mendelian disorders by means of the TruSight One Expanded Sequencing Panel Kit. The obtained sequencing reads were aligned to the NCBI human genome assembly (hg19) using the Burrows Wheeler Alignment Tool (BWA-MEM).

**P2:** The sample was processed on an ion Proton platform (Life Technologies). Approximately 33 MB of coding exons were covered by consensus coding sequences as described. The amplicon library was constructed using PCR-based targeted amplification using highly multiplexed primer pools.

**1.3 Variant annotation, filtering and prioritization**

**P1:** The variant calling (HaplotypeCaller) was performed according to GATK best practice guidelines (available at https://gatk.broadinstitute.org/hc/en-us) for calling single nucleotide variants, insertions and deletions. The evaluation of the called variants was performed using VarSeq software from Golden Helix® (Bozeman, Montana, USA). The exome-wide average read coverage was 95.5, whereas that of the multigene panel was 110. Variants were filtered based on minor allele frequency (MAF) using an in-house database, published mutations and based on the MAF listed in the Genome Aggregation Database (gnomAD) (MAF ≤ 0.5). The remaining variants were filtered for genes associated with HPO terms based on patient´s clinical symptoms (seizure, epilepsy) with predefined criteria, namely, read depth of > 9, current variant allele frequency of > 0.19 and Phred scale base quality score > 99. Six prediction tools for independent assessments of the potential pathogenicity of filtered missense variants (SIFT, Polyphen2 HVAR, MutationTaster, MutationAssessor, FATHMM, FATHMM MKL Coding) were used, and variants with at least 4 predictions as damaging/pathogenic/likely pathogenic were selected. Loss-of-function variants were independently considered for further analysis. All variants were assessed for their interpretation in the ClinVar database. Reported variants were classified based on the ACMG guidelines (Richards et al. 2015).

**P2:** After base calling and filtering out low-quality sequences, a standardized bioinformatics pipeline was applied to annotate the detected variants and selected genes with a potential influence on the described phenotype. The exome is estimated to cover approximately 2 % of the genome, but it contains at least 85 % of all currently identified disease-relevant mutations. Centogene uses a whole exome sequencing platform based on the Life Technologies platform. The following limitations apply to all analyses using next-generation sequencing (NGS): 1) NGS has a false-positive rate of 5-10%. Any clinically relevant variant identified by NGS is verified by Sanger sequencing (see below); 2) Due to technical limitations, WES cannot cover the entire exome. Statistical information on the coverage of the individual analysis is part of each report. To minimize the inherent limitations of WES analysis, we always work with a high degree of amplicon coverage (>100x) and require that >90% of all amplicons are covered at least 20x; 3) NGS is not reliable in repeat expansion regions. Therefore, NGS cannot provide data for the Huntington's disease genes, the majority of SCA genes, or myotonic dystrophy repeat regions. Furthermore, NGS cannot analyze copy number variants (CNVs). Centogene is continuously working on improving bioinformatics applications. This will increase the sensitivity and specificity of NGS and improve the evaluation and validation of difficult sequence regions and large structural rearrangements in a clinical setting. Statistical analysis: Number of mapped reads: 85,888,063; Percent reads on target: 93.11 %; Number of amplicons: 293,903; Average reads per amplicon: 272.1; Amplicons with at least 20 reads: 96.95 %. Reported variants were classified based on the ACMG guidelines (Richards et al. 2015).

**1.4 Sanger sequencing**

Both reported patient´s variants were confirmed by Sanger sequencing. Targeted testing of parental DNA to examine the origin of variants detected in the patient´s DNA was performed by Sanger sequencing. P2: The Sanger sequencing method used by Centogene is optimized for the confirmation and interpretation of relevant variants identified by WES. Sanger sequencing is performed in both forward and reverse directions. Novel pathogenic and likely pathogenic (LP) variants in the *PIGQ* gene have been submitted to ClinVar with accession numbers XXX and XXX (<https://www.ncbi.nlm.nih.gov/clinvar/submitters/279559/>).

**2 Results**

**2.1 Patient 1 - Case report**

Patient 1 (P1) was male, the first child of non-consanguineous parents from Austria. This first pregnancy was complicated by polyhydramnios diagnosed by ultrasound at the 26th week of gestation. He was born via cesarean section at 38 weeks of gestation. Apgar score was 9/9/9, and birth weight was 3420 g (50. – 75. percentile). He breathed spontaneously, but in the next few minutes, he developed cyanosis and was transferred to the neonatal intensive care unit. He presented with meteorism, diastasis of the musculus rectus abdominis, macroglossia, dysmorphic large ears, hepatosplenomegaly, and worsening respiratory status. Polysomnography resulted from sleep apnea, with oxygen saturation decreased to 80% with spontaneous adjustment. The patient did not need oxygen therapy, only during feeding from a bottle. Because of a polyglobulia, he needed infusion therapy for the first 4 days. The bilirubin level was 18 mg/dl, with the need for phototherapy. Due to facial dysmorphism, Beckwith-Wiedeman syndrome was suspected but genetically not confirmed. On day 13, he was released into home care with apnea monitoring. Aged 3 months, he was 59 cm long (25.-50. percentile) and weighed 5340 g (25.-50. percentile). The first seizure episode manifested as twitching of the right extremities with apnea. Mouth-to-mouth breathing by the mother was needed 10 times daily, mostly in series, independent from daytime. Interictal EEG at this time was without epileptic activity and appropriate for his age. MRI of the brain showed enlarged lateral ventricles but no other pathology in brain parenchyma. The metabolic investigation: ACC profile, lysosomal storage diseases (LSD) screening, amino acids, organic acids, VLCFA, and pipecolic acid were in normal ranges. Pyridoxine-dependent epilepsy was genetically excluded. Because of evident dysmorphic features, the genetic investigation continued by karyotyping with normal karyotype 46, XY, and by array CGH without any genomic imbalance. The investigation continued with a whole exome analysis (WES) without finding any causal variant. The cerebrospinal fluid investigation was negative. By polysomnography, the central and mixed sleep apnea with SatO2 decreased to 78 % with spontaneous adjustment and one episode with short bradycardia 50/min. was measured. At the age of 5 months, myoclonic seizures on the left side of the body appeared with the first symptoms of global developmental delay. USG brain with normal structures and normal EEG without epileptic activity were seen. Aged 6 months, his brain MRI showed enlarged lateral ventricles but no other pathology in brain parenchyma. At the age of 7 months, seizures with eye deviation and smacking movements started. Lamotrigine was ordered and well tolerated, but with less success. At the age of 8 months, he weighed 7200 g (3.-10. percentile), was 68 cm tall (10.-25. percentile) and had a head circumference of 44 cm (10.-25. percentile). There was no visual fixation and no visual contact, the eyelids could not close completely (lagophthalmos), and vertical nystagmus was noticed with normal photoreaction. The big deep set and dysmorphic ears with macroglossia were present. Neurological examination revealed weak spontaneous motor activity, muscular hypotonia, weak head control, and deep tendon reflexes. The TSH level was 8 µmol/ml (N: 0,35-3,5), fT3 8,8 pmol/l (N: 3,1-6,5), fT4 19,8 pmol/l (N: 10,3-21,9) evaluated as latent hypothyreosis. The level of ALP was elevated to 632 U/l (N: 82-383). The first examination of CK: 139 U/l (N:<170) was normal in time without infection. Vitamins, including B12, were in the normal range. ECG showed the first-degree AV block, PQ interval: 0,16 s (N: <0,14), ECHO cardiography showed aortal insufficiency, and recommended anti-endocarditis prophylaxis with antibiotics. EEG showed hypsarrhythmia during sleep without unambiguous sleep elements and a photostimulation frequency of 1/s without convulsive activity. Interictal, the frequent spikes and sharp waves in the left parietal-temporal (PT) region and ictal period were characterized by 20 times tonic seizures with upper limb extension and upturned eyes, a trigger point in the left PT region. Spikes-waves with generalization and fast rhythmic activity concluded the pathologic EEG interictal and ictal epileptic activity in the left PT region. Modified hypsarrhythmia with multifocal epileptogenic activity with absent normal age-appropriate basal activity. The therapeutic test with 200 mg pyridoxine was without reaction. After folic acid and pyridoxal phosphate, no evident PT focus and modified hypsarrhythmia stayed unchanged. USG of liver diagnosed enlargement +2 cm. MRI of the brain showed significant enlargement of the bifrontal subarachnoid space, severe myelination delay, and decreased volume of cerebral white matter. The therapy with Lamotrigine 5,2 mg/kg/day and Clobazam and intravenous pulse corticosteroid therapy by Dexamethason 20 mg/m2 3 days in 6 cycles/4 weeks pause caused fatigue and less seizure activity. The Pyridoxin 2x50 mg was added. At the age of 1 year, the sequencing and MLPA of the *PLP1* gene for Pelizeus-Merzbacher syndrome were negative. In the same way, the genes NCL1 and NCL2 associated with Neuronal ceroid lipofuscinosis type 1 and type 2 were tested with negative results. At 13 months, he weighed 6750 g (3. percentile) and was 67 cm tall (10. percentile). The Acylcarnitine profile was negative, but triacyl glycerides were high due to Dexamethason treatment without complications. Seizures persisted with a frequency every 2-5 days. WES focused on epilepsy was inconclusive. At the age of 3 and 5,5 years, the hyperCKemia during respiratory infection (> 5000U/L) was measured. At 9 years, WES data reevaluation focuses on epilepsy, and rhabdomyolysis has not recognized any disease-associated gene defect. At the age of 10 years, he weighed 16 kg (< 3. percentile), and the hyperCKemia during respiratory infection (> 5000U/L) (N: <170) was detected. Aged 10,5 years, the CK 180 U/l with CRP 3 under the upper respiratory tract infection was noticed.

At 11, the upper respiratory tract infection with fever (TT 40°C, CRP 19, and elevated CK: 939U/L) was diagnosed. At this time, the child got ill with gastroenteritis, and CK: 4943U/L and Myoglobin: 368 ng/ml (N: 0-9) were measured—possible differential diagnosis of L-Karnitin, ev. CTPII deficiency was also suspected. At age 11,5 years, his weight was 16 kg (< 3. percentile: -6,37 Z), and he got bronchitis with CK: 238U/L and later also by obstructive bronchitis, the elevated CK: 367U/L values were measured. The WES bioinformatic data reanalysis with the indication to use the HPO terms rhabdomyolysis/myopathy and suspicion of mitochondrial myopathy or hereditary metabolic disorder was indicated. Afterward, the recurrent pathogenic in-frame deletion c.1199_1201del (p.Tyr400del) and a novel likely pathogenic variant c.1092dupC (p.Phe365leufs*78) were found in the *PIGQ* gene. The trans-position of the variants confirmed the diagnosis of *PIGQ*-related glycophosphatidylinositol deficiency. Three months after the diagnosis was set, P1 received febrile infection with dehydration, prerenal failure, and metabolic acidosis, and the patient died at the age of 13 years due to asphyxia. At this time, the CK was 464 U/L.

**2.2 Patient 2 - Case report**

Patient 2 (P2) was male, the first child of non-consanguineous parents from Austria, from the first pregnancy complicated by polyhydramnios. The increased nuchal translucency was diagnosed (NT 3.4 cm). He was born spontaneously at 40+0 weeks of gestation, with a weight of 3 720 g (75. percentile), a length of 54 cm (50. percentile), and a head circumference of 38 cm (25. percentile). At birth, an Inborn error of metabolism due to initial symptoms such as an enlarged spleen and liver with normal liver parameters, low muscle tone, dysmorphic facial features, and gingival enlargement were suspected. In the first months of life, he presented jaundice and severe hypotonia (floppy infant), but with limb hypertonia and hyperextensibility of the whole body. He had feeding difficulties and needs with nasogastric tube. Later symptoms included global development delay, poor weight gain, regular vomiting, large low-set ears with large ear lobes, depressed nasal bridge, anteverted nares, pectus carinatum, lack of visual fixation, and seizures. He suffered from frequent upper respiratory tract infections, inspirations by eating, and obstructive sleep apnea syndrome, treated by non-invasive ventilation. Brain MRI, including spectroscopy, showed changes in the sense of a hepatic encephalopathy or a previous hypoxic event, even if neither was present. Enlargement of the left ventricle and normal myelinization of the dorsal internal capsule were confirmed. The perinatal EEG showed a conspicuous background activity without spasm potentials. Very early after the birth, abnormal movements with legs and arms appeared. Aged 1 year, seizures were present and needed antiseizure medication. He did not have any teeth at one year and did not develop any motor skills. A significant milestone was being able to be spoon-fed from six months, he would grip fingers or his own hands, but not toys, and the language was utterly absent. He had no hypothyroidism, and his CK levels were normal. The myoglobin has never been measured. None of the many tests conducted proved to be conclusive. They were given various potential diagnoses (Mukopolysacharidose Type II or IV, Gangliosidosis, Morbus Sandhoff, Morbus Wolmann), which were then ruled out either biochemically, clinically, or enzymatically. The storing of glycolipids and glycogen in various cells (sweat glands, muscle cells, fibroblasts, and macrophages) was confirmed. The enzyme analysis indicated Fucosidose, and simultaneously, whole exome sequencing (WES) identified the compound heterozygotes mutations in the *PIGQ* gene: the recurrent pathogenic in-frame deletion c.1199_1201del (p.Tyr400del) and novel VUS missence variant c.1370T>G (p.Leu457Arg). The trans position of both variants was confirmed by analysis in parents. Pathogenicity of the novel missense variant was proven by a functional study on CHO. P2 died at 12 months of life because of pneumonia.

**References**

Richards, S., Aziz, N., Bale, S., Bick, D., Das, S., Gastier-Foster, J., et al. (2015). Standards and guidelines for the interpretation of sequence variants: a joint consensus recommendation of the American College of Medical Genetics and Genomics and the Association for Molecular Pathology. *Genet in Med: official journal of the American College of Medical Genetics,* 17(5), 405–424. [doi:10.1038/gim.2015.30](https://doi.org/10.1038/gim.2015.30)

**Supplementary materials (Figures)**


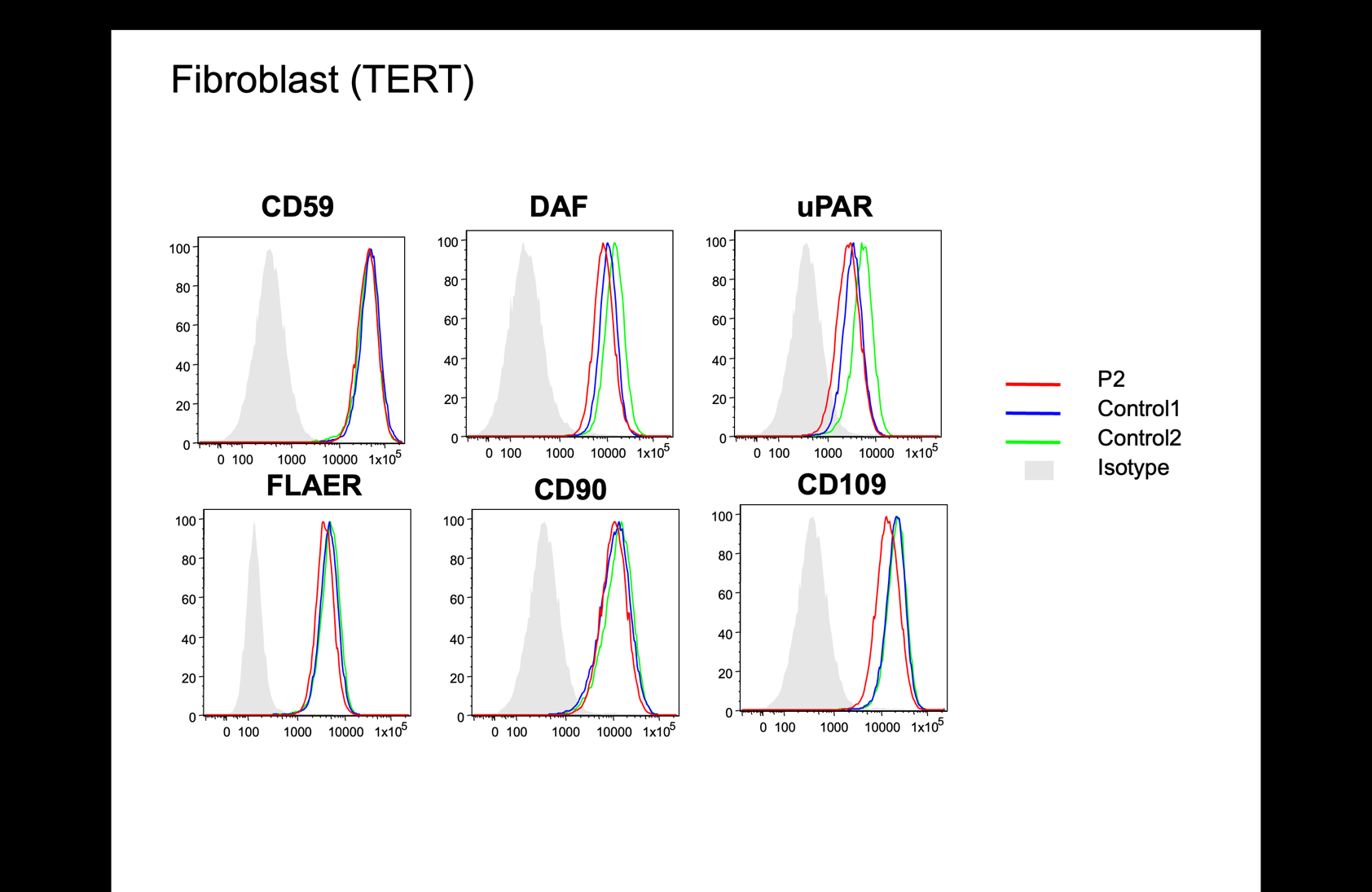


**Supplementary Figure 1.** **Fibroblasts (TERT).** We have immortalized the fibroblasts by telomerase reverse transcriptase (TERT) using retrovirus and stained with antibodies of various GPI-APs. Expression of uPAR and CD109 on patient’s fibroblasts is slightly reduced.


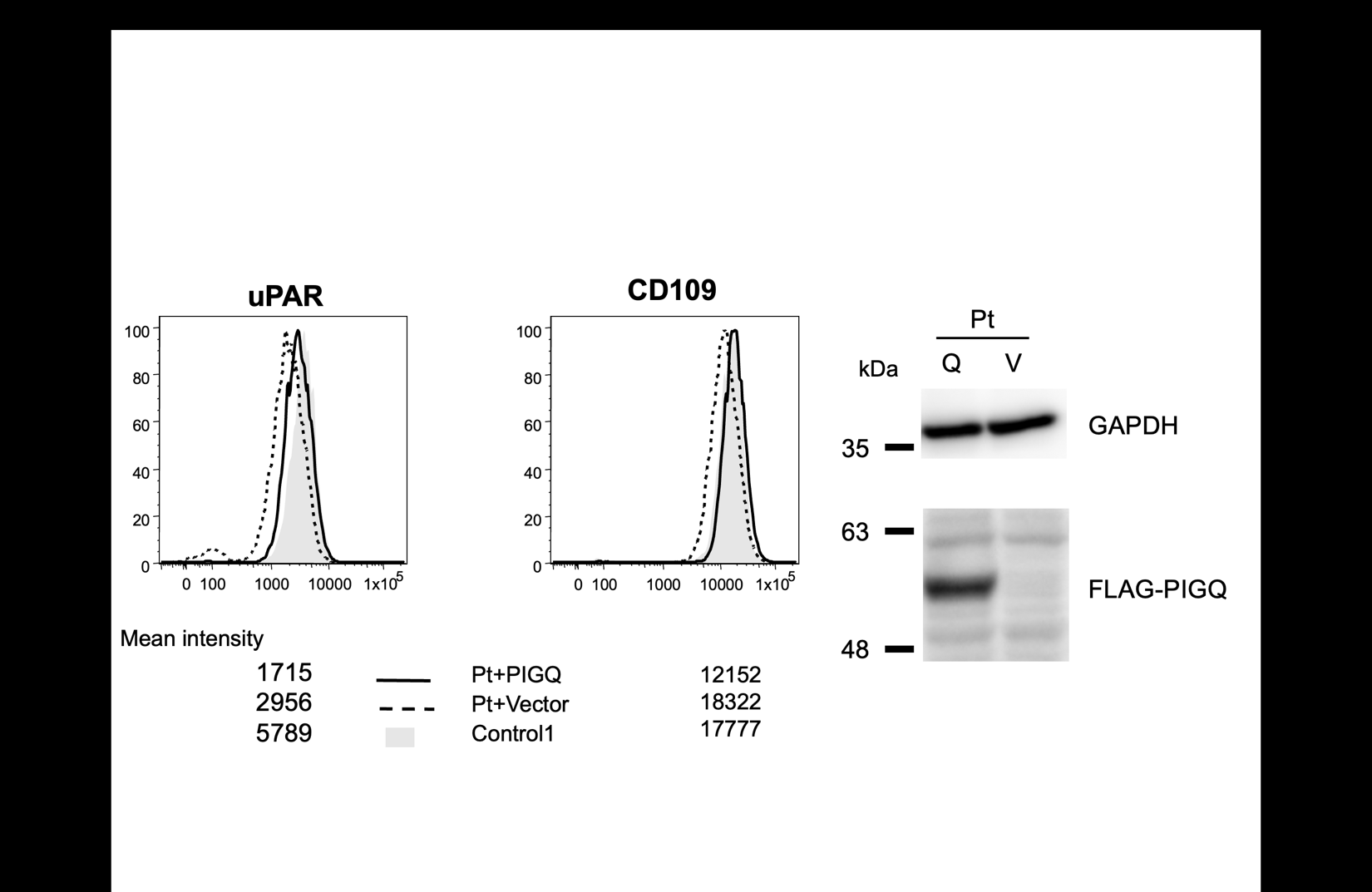


**Supplementary Figure 2.** **Fibroblasts.** To confirm that the reduction is caused by PIGQ deficiency, we transduced wild type PIGQ cDNA bearing hygromycin resistant gene and made the permanent transfectants. Patient’s fibroblasts expressing wild type hPIGQ restored the surface expression of GPI anchored protein to the control level while the empty vector transfectants did not. Lysates were applied to SDS-PAGE, and western blotting was performed using an anti-FLAG antibody (M2, Sigma, St. Louis, MO) to detect PIGQ expression and anti-GAPDH (6C5, Life Technologies, CA) to detect endogenous GAPDH for loading control.


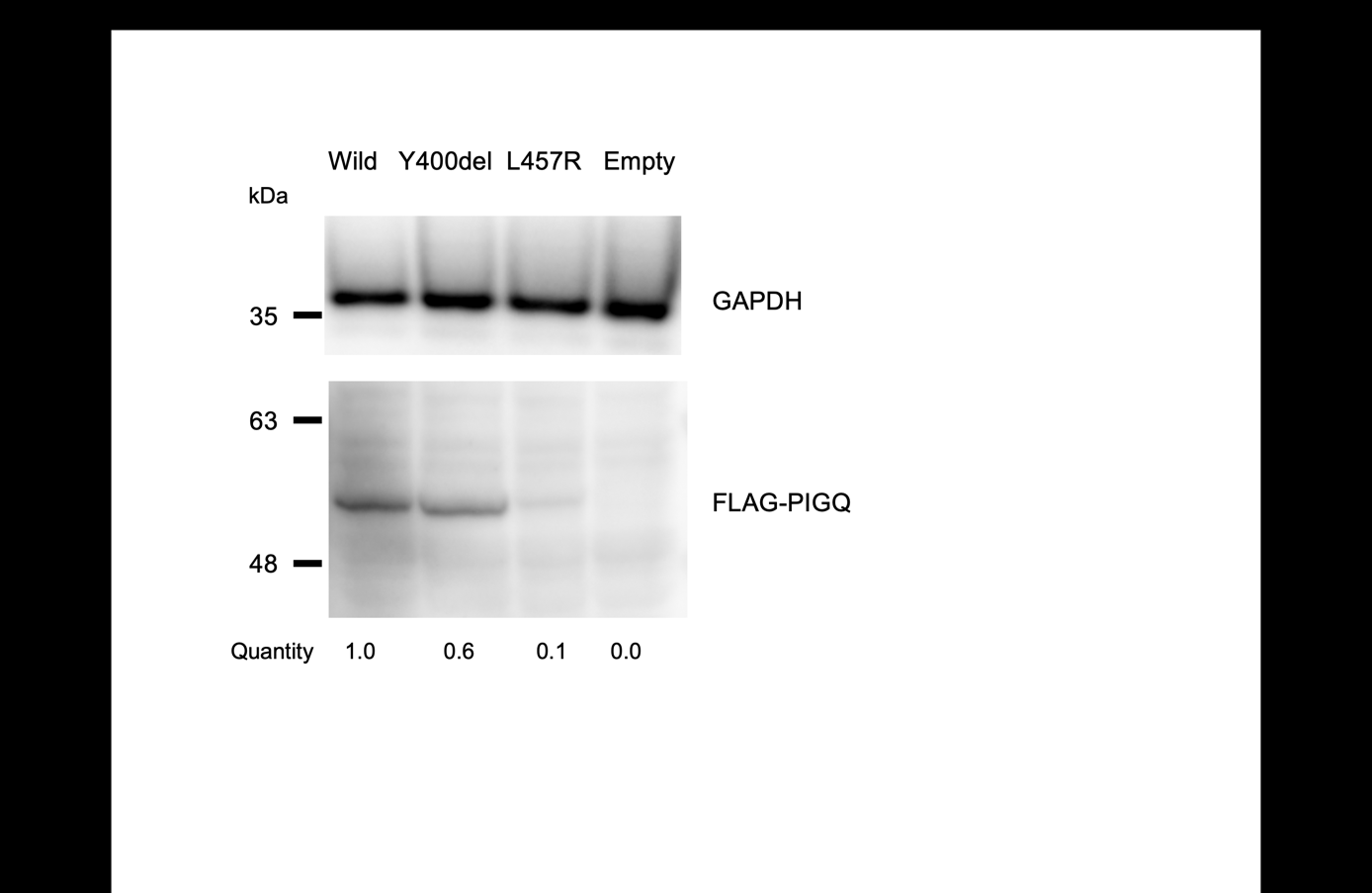


**Supplementary Figure 3. Western blot.** We transiently transfected the wild type or mutant pME FLAG PIGQ in HEK 293 cells. Two days later, lysates were applied to SDS-PAGE, and western blotting was performed using anti-FLAG antibody (M2, Sigma, St. Louis, MO) to detect PIGQ expression and anti-GAPDH (6C5, Life Technologies, CA) to detect endogenous GAPDH for loading control. Luciferase activities monitored transfection efficiency using Luciferase assay kit (Promega, Madison, WI). Quantification was performed by normalizing the band intensities of FLAG-PIGQ to those of GAPDH (as a loading control) and further adjusting for transfection efficiency using luciferase activity.

**Supplementary Figure 4. The Sanger sequencing chromatograms confirming the *PIGQ* gene variants in Patient 1 (P1).** A,B) Whole exome sequencing identified compound heterozygous *PIGQ* variants, c.1092dupC, p.(Phe365Leufs*78) and c.1199_1201del, p.(Tyr400del). C,D) Both variants were confirmed with Sanger sequencing in the patient depicted in sequencing chromatograms. Frameshift variant c.1092dupC; p.Phe365Leufs*78 (NM_004204.3, hg19) was paternally and inframe variant c.1199_1201del; p.Tyr400del (NM_004204.3, hg19) maternally inherited.


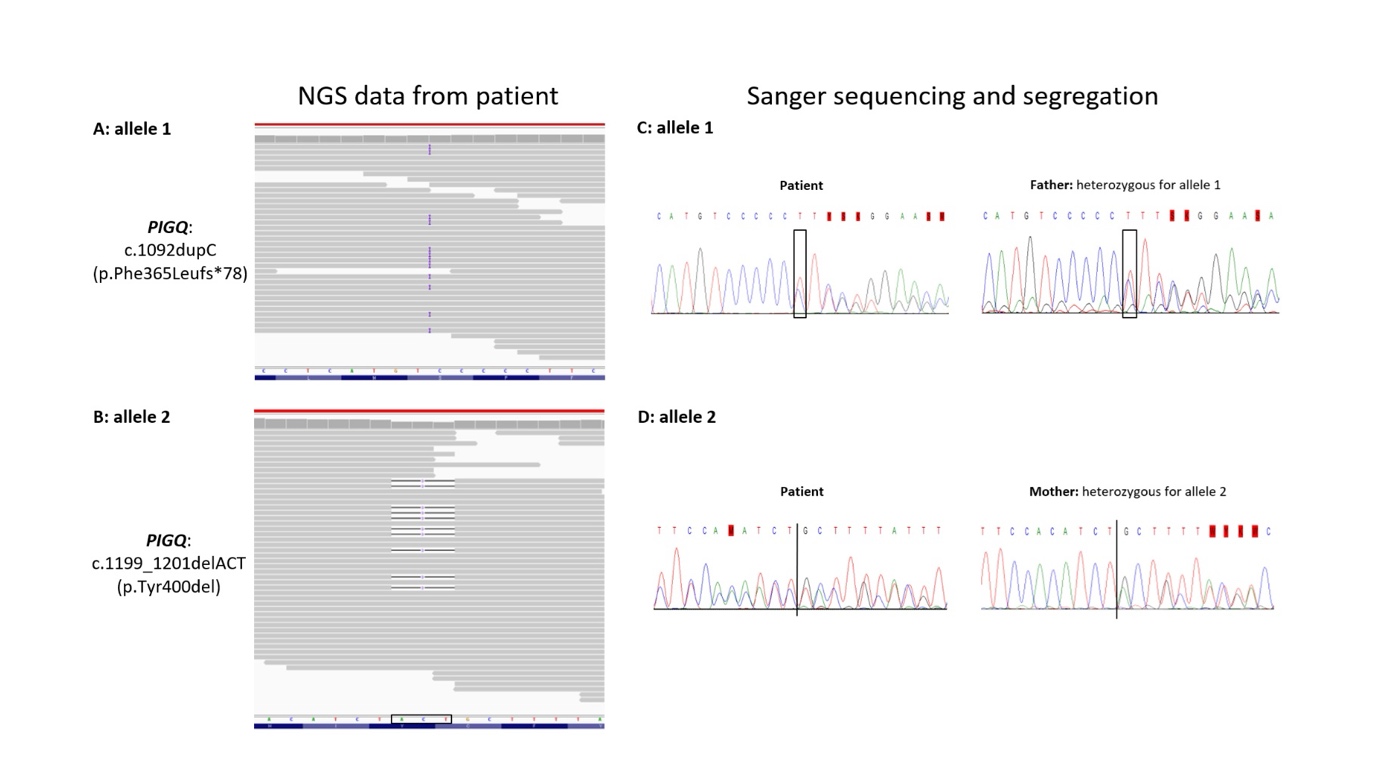


**Supplementary Figure 5. The Sanger sequencing chromatograms confirming the *PIGQ* gene variants in Patient 2 (P2).** Whole exome sequencing identified compound heterozygous *PIGQ* variants, c.1370T>G, p.(Leu457Arg) and c.1199_1201del, p.(Tyr400del). Both variants were confirmed with Sanger sequencing in the patient depicted in sequencing chromatograms. A) Missense variant c.1370T>G; p.Leu457Arg (NM_004204.3, hg19) was maternally, and B) in frame variant c.1199_1201del; p.Typ400del (NM_004204.3, hg19) paternally inherited.


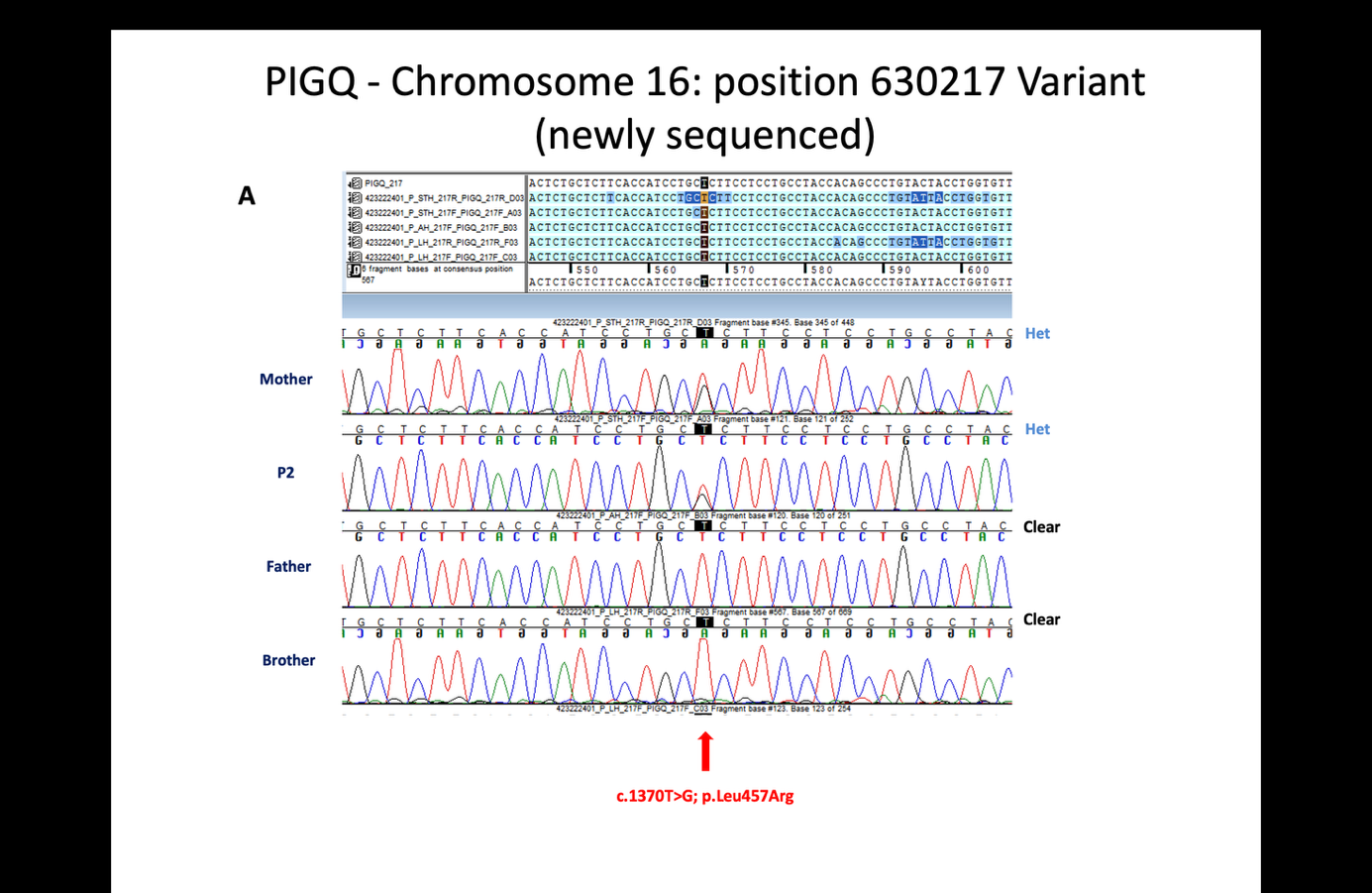


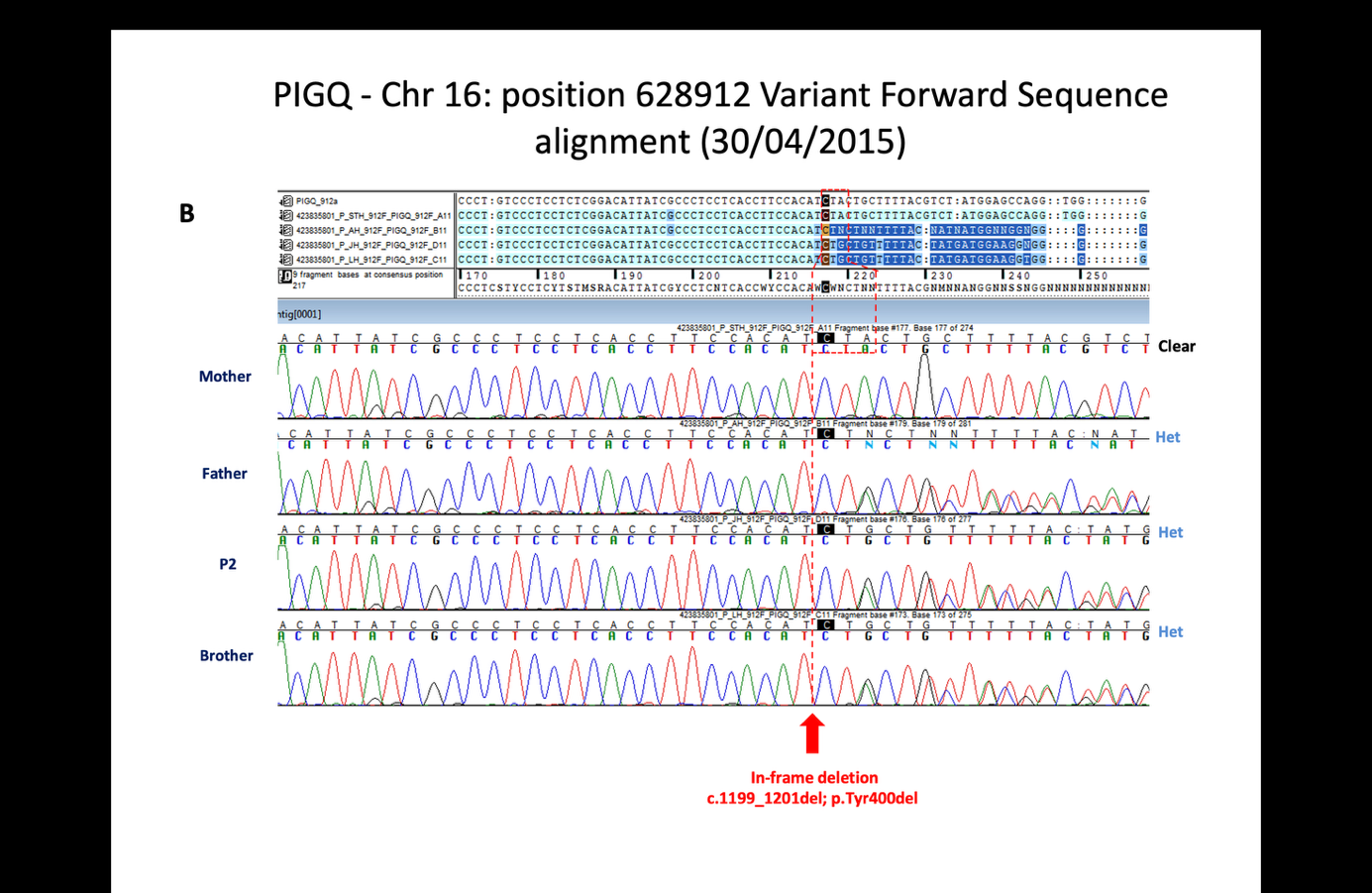

Supplement: Supplementary file 9 [file DataSheet1.docx]
